# Mechanically tuned 3 dimensional hydrogels support human mammary fibroblast growth and viability

**DOI:** 10.1186/s12860-017-0151-y

**Published:** 2017-12-16

**Authors:** Kathryn Woods, Catlyn Thigpen, Jennifer Peyling Wang, Hana Park, Abigail Hielscher

**Affiliations:** 1 Department of Pathology and Microbiology, University of Nebraska Medical Center, 985900 Nebraska Medical Center, Omaha, NE 68198 USA; 2 Department of Biomedical Sciences, Georgia Philadelphia College of Osteopathic Medicine, Suwanee, GA 30024 USA; 3 Anschutz Medical Campus Skaggs School of Pharmacy, University of Colorado, 12850 E. Montview Blvd, Aurora, CO 80011 USA

**Keywords:** 3 dimensional hydrogels, Microbial transglutaminase, Myofibroblasts, Human mammary fibroblasts, Extracellular matrix

## Abstract

**Background:**

Carcinoma associated fibroblasts (CAFs or myofibroblasts) are activated fibroblasts which participate in breast tumor growth, angiogenesis, invasion, metastasis and therapy resistance. As such, recent efforts have been directed toward understanding the factors responsible for activation of the phenotype. In this study, we have investigated how changes in the mechanical stiffness of a 3D hydrogel alter the behavior and myofibroblast-like properties of human mammary fibroblasts (HMFs).

**Results:**

Here, we utilized microbial transglutaminase (mTG) to mechanically tune the stiffness of gelatin hydrogels and used rheology to show that increasing concentrations mTG resulted in hydrogels with greater elastic moduli (G’). Upon encapsulation of HMFs in 200 (compliant), 300 (moderate) and 1100 Pa (stiff) mTG hydrogels, it was found that the HMFs remained viable and proliferated over the 7 day culture period. Specifically, rates of proliferation were greatest for HMFs in moderate hydrogels. Regarding morphology, HMFs in compliant and moderate hydrogels exhibited a spindle-like morphology while HMFs in stiff hydrogels exhibited a rounded morphology with several large cellular protrusions. Quantification of cell morphology revealed that HMFs cultured in all mTG hydrogels overall assumed a more elongated phenotype over time in culture; however, few significant differences in morphology were observed between HMFs in each of the hydrogel conditions. To determine whether matrix stiffness upregulated expression of ECM and myofibroblast markers, western blot was performed on HMFs in compliant, moderate and stiff hydrogels. It was found that ECM and myofibroblast proteins varied in expression during both the culture period and according to matrix stiffness with no clear correlation between matrix stiffness and a myofibroblast phenotype. Finally, TGF-β levels were quantified in the conditioned media from HMFs in compliant, moderate and stiff hydrogels. TGF-β was significantly greater for HMFs encapsulated in stiff hydrogels.

**Conclusions:**

Overall, these results show that while HMFs are viable and proliferate in mTG hydrogels, increasing matrix stiffness of mTG gelatin hydrogels doesn’t support a robust myofibroblast phenotype from HMFs. These results have important implications for further understanding how modulating 3D matrix stiffness affects fibroblast morphology and activation into a myofibroblast phenotype.

**Electronic supplementary material:**

The online version of this article (10.1186/s12860-017-0151-y) contains supplementary material, which is available to authorized users.

## Background

Breast cancer is one of the leading causes of cancer-related deaths among women worldwide. Albeit dismal, the mortality rate of breast cancer has diminished due to the availability of treatments including total or partial removal of the breast, chemotherapy and radiation therapy, as well as endocrine and targeted therapies. Despite these options, the preponderance of recurring cancer and metastasis still remain [1]. While the growth and metastatic potential of breast cancer is dependent on a number of factors, the tumor microenvironment which includes fibroblasts, immune cells, endothelial cells and the extracellular matrix (ECM), have been reported to play a role in these tumor cell behaviors [2]. Of particular interest is the role of activated fibroblasts, referred to as myofibroblasts or cancer associated fibroblasts (CAFs), in breast tumor progression and metastasis.

Myofibroblasts are activated fibroblasts which have known roles in wound healing, tissue morphogenesis, fibrotic diseases and tumorigenesis [3–5]. First described in granulation tissues, myofibroblasts are highly contractile and accordingly express alpha smooth muscle actin (α-SMA) in actin stress fibers [6]. Additionally, myofibroblasts secrete several growth factors and cytokines of which transforming growth factor beta (TGF-β1) is the most well-known [4]. Furthermore, myofibroblasts contribute to the desmoplastic reaction, a feature of breast carcinomas [7], as a result of their increased expression and deposition of a number of ECM proteins [8]. With regard to a role in tumorigenesis, these activated stromal cells are termed carcinoma associated fibroblasts (CAFs). Similar to myofibroblasts, CAFs exhibit increased contractile capabilities and marked up-regulation of α-SMA [5]. CAFs have also been reported to express the smooth muscle marker desmin and the cell surface glycoprotein fibroblast activation protein (FAP) [5, 9]. In addition, CAFs have been documented to secrete several growth factors including TGF-β1 [10, 11], and stromal derived factor 1 (SDF1) [12] amongst others and have further been reported to remodel the ECM as a result of their deposition of new ECM proteins and expression of matrix degrading enzymes such as matrix metalloproteinases (MMPs). Within breast tumors, CAFs have been reported to comprise up to 80% of the fibroblasts within the tumor microenvironment [13]. Given the prevalence of these cells in breast tumors, there is much interest devoted to better understanding the factors which not only drive the acquisition of the CAF phenotype but how these CAFs contribute to aberrant epithelial cell behavior and breast tumor progression.

One mechanism responsible for the conversion of fibroblasts into myofibroblasts or CAFs is matrix stiffness or increased tissue rigidity [14–16]. The altered mechanical properties of tissues are in large part a result of changes in the architecture of the ECM in which increased deposition and aberrant cross-linking of matrix proteins accompanies pathologic conditions such as breast cancer. In 2D cultures, matrix stiffness has been shown to promote the transition of non-activated fibroblasts to myofibroblasts [17]. For example, Peyton et al. [18] cultured smooth muscle cells atop mechanically tuned poly(ethylene) glycol (PEG) hydrogels functionalized with cell adhesive binding sites. Matrix stiffness combined with different cell adhesive sites promoted cell spreading, proliferation and focal adhesions and F-actin fiber formation, properties which are indicative of an activated fibroblast phenotype [18]. Similarly, Li et al. [15] demonstrated that portal fibroblasts differentiated to myofibroblasts following culture atop mechanically stiff polyacrylamide gels. More recently, Liu et al. [19] reported that lung fibroblasts cultured atop mechanically tuned polyacrylamide gels exhibited enhanced proliferation and mRNA expression of collagens I and III with increasing matrix stiffness [19]. While these and other results clearly point to a role for matrix stiffness in the differentiation of fibroblasts to myofibroblasts in 2D, further analysis of this phenomenon in 3D is necessary.

Increasingly, the use of 3D culture approaches have become desirable as these systems better replicate the in-vivo environment and as a result, provide a platform from which changes in cell behavior may be more accurately addressed. With regard to a role for mechanical stiffness in the activation of a myofibroblast phenotype in 3D cultured cells, Galie et al. [20] reported that low serum concentration in cooperation with increased matrix stiffness of collagen gels promoted the myofibroblast transition of cardiac fibroblasts. In addition, Karamichos et al. [21] utilized mechanically constrained, partially constrained and unconstrained collagen gels to show that corneal fibroblasts seeded in constrained matrices exhibited enhanced alignment, spreading and contractile forces, properties associated with a myofibroblast phenotype [6]. Indeed, these studies indicate that the mechanical properties of 3D hydrogels may not only be tuned, but exploited to investigate cell behavior.

In the present study, we have utilized a gelatin based hydrogel to investigate the role of mechanical stiffness on the acquisition of a myofibroblast phenotype in 3D encapsulated HMFs. Gelatin, a natural polymer produced via collagen hydrolysis, is an ideal scaffold because of its biocompatibility, ease with which to adjust its mechanical properties [22], and its derivation from collagen, a principal component of the interstitial matrix [23] and of breast tissue [24]. These characteristics combined not only make gelatin an optimal cellular scaffold for HMF growth, but for growth of several types of mammalian cells. To manipulate the mechanical stiffness of the gelatin hydrogel, microbial transglutaminase (mTG) was used. Derived from *Streptoverticillium mobaraense*, mTG is desirable as the enzyme is stable over a wide temperature and pH range and crosslinks lysine and glutamine residues in collagen and gelatin hydrogels, enabling fine-tuned control over the mechanical strength of the hydrogel [25]. Previous reports have shown that fibroblasts are viable and proliferate following encapsulation in mTG cross-linked hydrogels [26]. Additionally, studies by Yung et al. [25] found that mTG cross-linked gelatin hydrogels supported the growth of HEK 293 human embryonic kidney cells.

In an effort to better understand the role of 3D matrix stiffness on HMF properties, we utilized mTG to tune the mechanical stiffness of gelatin hydrogels and assessed how differences in matrix stiffness influenced HMF acquisition of a myofibroblast phenotype. Here, we report that a range of concentrations of mTG yielded hydrogels with various mechanical stiffnesses and further demonstrated that HMFs encapsulated in compliant, moderate and stiff hydrogels were viable and proliferated during the culture period. Furthermore, markers of myofibroblasts were assessed and varied in expression during both the culture period and according to matrix stiffness. Overall, these results have important implications for understanding how 3D matrix stiffness affects fibroblast properties and acquisition of myofibroblast-like features, findings which have important implications for understanding the role of tumor stiffness and its effects on cell behavior.

## Methods

### Reagents and antibodies

Reagents used include microbial transglutaminase (ACTIVA TI; Ajinomoto, Tokyo, Japan) and porcine gelatin (Sigma, Allentown, PA). The dilutions and suppliers for the antibodies used are provided in Table 1.

**Table 1:** Antibodies. Dilutions and manufacturer information for antibodies used for western blot experiments

Reagents	Manufacturer	Dilution
Rabbit anti-human α-SMA	Abcam	1:150-300
Mouse anti-human vimentin	Abcam	1:500
Rabbit anti-human fibronectin	Sigma	1:400
Rabbit anti-human collagen	Gift: Larry Fisher, NIH	1:2000
Rabbit anti-human collagen IV	Abcam	1:200-250
Rabbit anti-human laminin	Abcam	1:400
Rabbit anti-human GAPDH	Cell Signal Technologies	1:2000

### Isolation of microbial transglutaminase

Microbial transglutaminase (mTG) was purified based on methods described by Kuwahara et al. [27]. A 5% solution of mTG was made in 50 ml of Buffer A (20 mM phosphate and 2 mM EDTA, pH 6.0). Initially, 2.5 ml of Sepharose FF beads (GE Healthcare, Atlanta, GA) were allowed to settle out of solution. The supernatant was removed and the beads were washed with 5 column volumes of Buffer A. The mTG solution was added to the pre-equilibrated Sepharose beads and incubated overnight at 4 **°**C at constant shaking. The protein and bead mixture was loaded into a column and washed 4× with Buffer A. A volume of 5 ml of 200 mM NaCl prepared in Buffer A was added to the column to collect fractions of mTG. The NaCl was removed and the protein was concentrated using 10 kDa ultracentrifugal spin columns. Purity of the mTG was examined using a Coomassie stain. UV spectrometry was used to determine protein concentration. The mTG was stored at −80 **°**C for later use.

### Preparation of mTG-treated 3D Gelatin Hydrogels

A 7.5% solution of gelatin was prepared in 1X PBS. Once dissolved, the gelatin solution was heated at 37 °C for 1-3 h to liquefy then sterile filtered using a 50 ml steriflip equipped with a 0.22 μM filter. The filtered gelatin was stored at 4 °C. To cross-link gelatin, 7.5% gelatin was mixed with various concentrations of mTG (20-60 μg/ml) and plated in 24 well plates. mTG treated gelatin hydrogels were allowed to polymerize at 37 °C for 30–45 min.

### Thermal stability of mTG Hydrogels

To evaluate the thermal stability of mTG gelatin, 7.5% gelatin was cross-linked at 37 °C with the 20, 30 and 60 μg/mL of mTG. Upon cross-linking, the hydrogels were weighed to obtain the initial weight then incubated in 1 mL of 1X PBS at 37 °C. At each hour for a total of 5 h, the PBS was carefully removed from the gels and the gels were weighed. After weighing, the PBS was added back to the gels for continued incubation at 37 °C.

### Rheology of mTG Hydrogels

Measurements of the elastic moduli (G’) of 20, 30, and 60 μg/mL mTG cross-linked 7.5% gelatin hydrogels were obtained using a constant strain rheometer with a steel plate geometry (Ares RFS III Rheometer, TA Instruments). Prior to analyses, mTG hydrogels were allowed to polymerize at 37 °C for 35–45 min. Following polymerization, mTG cross-linked hydrogels were incubated overnight in 1X PBS at 37 °C to mimic cell culture conditions. The next day, the PBS was carefully removed and the gels were analyzed for differences in the elastic moduli. The strain was maintained at 10% and the frequency used was 10 Hz. All measurements were recorded as the stress/strain where stress is the force (N/m^2^) applied and strain is the relative deformation of the gel in response to the applied force. The measurement of stiffness was recorded in Pascals. Results are reported from duplicate samples for each tested mTG concentration.

### Cell culture

Human mammary fibroblasts (HMFs), an immortalized fibroblast cell line derived from a reduction mammoplasty, were obtained from Dr. Parmjit Jat of the University College London [28]. HMFs were cultured in DMEM (Thermo-Fisher, Waltham, MA) supplemented with 10% vol/vol heat inactivated FBS (Thermo-Fisher). Media was replaced every 2-3 days and cells were passaged after reaching 80-90% confluency using 0.25% trypsin EDTA (Sigma). HMFs were maintained at 37 °C in a humidified atmosphere containing 5% CO_2_.

### mTG treatment of 2D HMFs

To determine whether mTG was toxic to HMFs, HMFs were treated with several concentrations of mTG, ranging from 6.3 μg/mL to 500 μg/mL. Prior to administering mTG, 25,000 viable HMFs were plated in a 24 well plate and allowed to incubate overnight. Cell viability was determined using the trypan blue exclusion assay. Analyses were conducted using an automated cell counter (Thermo-Fisher). The next day, the cells were treated in triplicate with various concentrations of mTG and were left to incubate overnight at 37 °C. HMF viability was measured using a 1:10 dilution of the Alamar Blue (Thermo-Fisher) reagent. Absorbance readings were measured at a wavelength of 570 nm using a Synergy HT spectrophotometer (Biotek, Winooski, VT).

### 3D HMF encapsulation in Gelatin Hydrogels

To encapsulate HMFs in gelatin gels, 35,000 viable HMFs were re-suspended in 20 μL of serum-free DMEM. To this, 250 μL of gelatin containing 20, 30, or 60 μg of mTG was added to the cells. The gel encapsulated cell suspension was added to each of 4-10 wells of a 24-well plate. After plating, a gelation time of 30–45 min in a 37 °C incubator was given to enable crosslinking. Upon polymerization, 1 mL of complete media was added to each gel. The hydrogels were maintained at 37 °C in a humidified atmosphere containing 5% CO_2_ for varying time points over a 7-day incubation period.

### HMF viability in mTG Hydrogels

The Live/Dead viability assay (Thermo-Fischer) was used to quantify the percentage of viable HMFs found within the various mTG cross-linked hydrogels. HMFs were encapsulated at a density of 35-50,000 cells/well in gelatin hydrogels crosslinked with 20, 30 and 60 μg mTG. Viability was measured at days 1, 3, 5, and 7 post-encapsulation. Calcein AM (Thermo-Fisher) and ethidium bromide (Thermo Fisher) were used at a concentration of 1 μM each. Both reagents were mixed with 5 ml of pre-warmed 1X PBS and added to cell-encapsulated gels at a volume of 500 μL. The gels were incubated in the dark at room temperature for 45 min prior to imaging. The EVOS microscope (Thermo-Fisher) was used for imaging live and dead cells. A 10× objective was used to capture images in the GFP (live), RFP (dead), and phase channels. Three sets of images were taken per well at three non-overlapping locations. Images were analyzed using ImageJ (NIH) software, which enabled quantification of live and dead cells. Here, images representing live and dead cells were converted to binary images. Viable and nonviable cells were manually counted in quadrants assigned to each image.

### HMF morphology in mTG Hydrogels

To determine changes in morphology of HMFs encapsulated in the various hydrogels, ImageJ (NIH) was used to quantify cell circularity. Here, the circularity formula uses 4π*area/perimeter^2^ to designate a perfectly circular shape with a value of 1.0 and an elongated shape with a value of 0.0. To define the borders of a cell, the freehand polygon tool was used to outline the cell periphery. Next, “Measure” was selected to obtain the circularity results. Cells in which borders couldn’t be clearly defined or were found on the edges weren’t used in these analyses. Six images were analyzed in each of the hydrogel conditions and at each time point assessed (e.g. days 1, 3, 5 and 7). Three to seven cells were analyzed per image.

### HMF proliferation in mTG Hydrogels

HMFs were encapsulated in 7.5% gelatin gels cross-linked with 20, 30, or 60 μg/ml of mTG as previously described. Cell proliferation was assessed in triplicate on days 1, 3, 5, and 7 during the incubation period and was evaluated using the WST-1 assay (Roche; Mannheim, Germany) according to the manufacturer’s instructions. Briefly, the WST reagent was added directly to cell culture media in a 1:10 dilution. HMF encapsulated gels were allowed to incubate in WST reagent for 3 h in a humidified atmosphere at 37 **°**C and 5% CO_2_. Results were evaluated at a wavelength of 450 nm using a Synergy HT spectrophotometer. These assays were performed twice on triplicate samples for each of the gel conditions and time points. To account for any influence on the absorbance reading from the gel itself, the WST-1 assay was performed on control gels, lacking HMFs, cross-linked with 20, 30, and 60 μg/ml of mTG. The absorbance values from control gels were obtained, averaged, and then subtracted from the absorbance values of the gels with encapsulated HMFs.

### Weight measurements of HMF encapsulated Hydrogels

HMFs were encapsulated at a density of 147,000 cells per 35 mm dish in gelatin hydrogels cross-linked with 20, 30, or 60 μg of mTG. Following polymerization, 2 ml of complete media was added to the HMF encapsulated hydrogels and these were left in the incubator at 37 °C. After hydrogel swelling (1.5-2 h, time 0) and after 7 days of growth, the media was carefully removed and the HMF encapsulated hydrogels were placed in the -80C for 2 h to overnight then placed in a freeze drier for a period of 3-5 days. Hydrogels were subsequently weighed to determine whether any weight changes in the hydrogels were apparent at the end of the culture period.

### Enzymatic digestion of HMF encapsulated Hydrogels

HMFs encapsulated in 7.5% gelatin hydrogels cross-linked with 20, 30, or 60 μg/ml of mTG were subjected to enzymatic digestion. Analyses were conducted at days 1, 3, 5 and 7 of culture. After removing the media, the gels were washed 2× with 1X PBS and treated with 0.5 mg/ml collagenase (Sigma-Aldrich) in DMEM containing a 1:100 dilution of protease inhibitor cocktail (Sigma-Aldrich). The gels were incubated for 90–105 min at 37 **°**C in a humidified atmosphere containing 5% CO_2_. Upon digestion, the contents of each well were combined and mixed with an equal volume of DMEM plus 10% FBS. The resulting mixture was centrifuged at 4500 RPM for 10 min. The supernatant was decanted and 300-500 μl of ice cold RIPA buffer, (150 mM NaCl, 1.0% triton X, 0.1% SDS, 50 mM Tris (pH 8.0), and 0.5% sodium chlorate) containing a 1:100 dilution of protease inhibitor cocktail was added to the pellet. The pellet was lysed on ice for 10 min and centrifuged at 12000 RPM for 10 min. The lysate was collected and stored at −80 **°**C. A DC (Bio-Rad) assay was used according to the manufacture’s protocol to measure protein concentration. Bovine serum albumin (BSA, Bio-Rad) was used to establish a standard curve.

### Western blot

HMFs isolated using collagenase digestion was analyzed for protein expression using western blot. HMFs were analyzed from 20, 30 and 60 μg/ml mTG gels at days 1, 3, 5 and 7 of culture. A total concentration of 10-25 μg of protein was prepared in laemmli buffer (Bio-Rad), and betamercaptoethanol (Sigma). RIPA buffer was added to reach a final volume of 30 μl/well. The LiCOR molecular weight marker (LiCOR, Lincoln, NE) was used as the ladder. The protein samples were heated at 95 **°**C for 5 min and loaded into each well of 4-20% tris-glycine gels (Bio-Rad). The gels were allowed to run for 30–45 min at 200 V. Using the Trans-Blot Turbo Transfer System (Bio-Rad), the proteins were transferred from the gels to PVDF membranes (Bio-Rad). The membranes were subsequently blocked in Odyssey blocking buffer (LiCOR) at room temperature under constant shaking for 2–3 h. The membranes were incubated with continuous shaking at 4 **°**C in the following primary antibodies: vimentin, α-SMA, fibronectin, collagen I, collagen IV and GAPDH (Table 1). The membranes were washed 3× for 15 min in washing buffer containing 0.1% tween 20 in 1X PBS. The membranes were then incubated at room temperature for 30 min in a 1:10,000 dilution of anti-mouse and/or anti-rabbit fluorescent secondary antibodies (LiCOR) at constant shaking. The membranes were subsequently washed in washing buffer 3× for 15 min at constant shaking and imaged using a 700 nm channel on the Odyssey CLx fluorescent imager (LiCOR). To quantify protein expression, the mean grey intensity of each band was analyzed using ImageJ. Here, a region of interest (ROI) was drawn around the largest band for a given protein within a row. This ROI was then used to measure the mean grey intensities in the remaining bands for the same protein. The pixel density of the protein bands and background was inverted by subtracting the mean grey values from 255. To normalize, the inverted density of the proteins of interest was divided by the averaged inverted density of GAPDH to obtain a ratio of expression. All proteins were analyzed from 2 to 4 blots each.

### TGF-β analyses

HMFs were encapsulated in 20, 30, 60 μg of mTG gelatin hydrogels and incubated for 1, 3, 5, and 7 days, during which time the media was not changed. At the end of each incubation period, conditioned media was collected and spun down at 1200 RPM to remove any cellular debris. The supernatant was collected and stored at −80 **°**C. TGF-β in the conditioned media was evaluated in triplicate samples and analyzed utilizing the Quantikine ELISA (RnD Systems) according to the instructions supplied by the manufacturer. The optical density was then evaluated at wavelengths of 450 nm using a Synergy HT Spectrophotometer. The control, which was cell culture media, was analyzed and subtracted from the experimental samples.

### Gel contraction assays

Gel contraction assays were set up as described by Duru et al. [29]. In brief, HMFs were encapsulated at a density of 147,000 cells in 1 ml of gelatin cross-linked with either 20, 30 or 60 μg/ml of mTG. The HMF/gelatin-mTG mixtures were plated in 35 mM dishes and a total of 3 hydrogels per mTG condition were prepared. Following polymerization, 2 ml of complete media was added to each hydrogel and these were incubated at 37 °C/5% CO_2_ for 48 h after which time, the gels were gently released from the sides of the dish using a sterile pipette tip (time 0). HMF encapsulated hydrogels were evaluated using a transilluminator at time 0 (Biorad, Chemidoc XRS) then visibly observed for changes in diameter during the remainder of the 7 day culture period.

### Statistical analyses

Statistical Analysis was performed using Prism from GraphPad (GraphPad Software, LaJolla, CA). One-way ANOVA followed by Turkey’s posttest and students ttests were utilized to analyze statistical significance. Two-way ANOVA followed by Bonferroni post-test was used to analyze significance between groups. For reporting significant difference: *p ≤ 0.05, **p ≤ 0.01, and ***p ≤ 0.001. Data are reported ±SD.

## Results

### HMF viability following 2D culture treatment with mTG

**Fig. 1:**
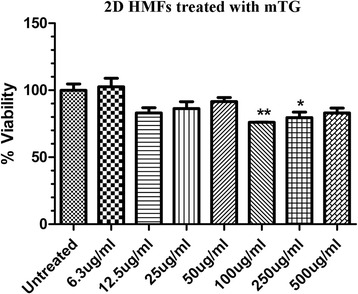
HMF responses to mTG in 2D culture. HMFs were treated in 2D with various concentrations of mTG. Up to 50 μg/ml of mTG, viability wasn’t significantly different from control, untreated cells. ^*^ *p* ≤ 0.05; ^**^ *p* ≤ 0.01; ^***^ *p* ≤ 0.001

Prior to encapsulation of HMFs in mTG crosslinked gelatin hydrogels, it was necessary to first determine whether the enzyme was toxic to HMFs in 2D cultures. As such, HMFs were treated in 2D with various mTG concentrations (6.3-500 μg /mL). After 24 h of treatment, the percent cell viability was measured. It was determined that HMFs were viable at most mTG concentrations tested, suggesting that overall the enzyme is non-toxic to HMFs (Fig. 1).

### Rheology of mTG hydrogels

**Fig. 2:**
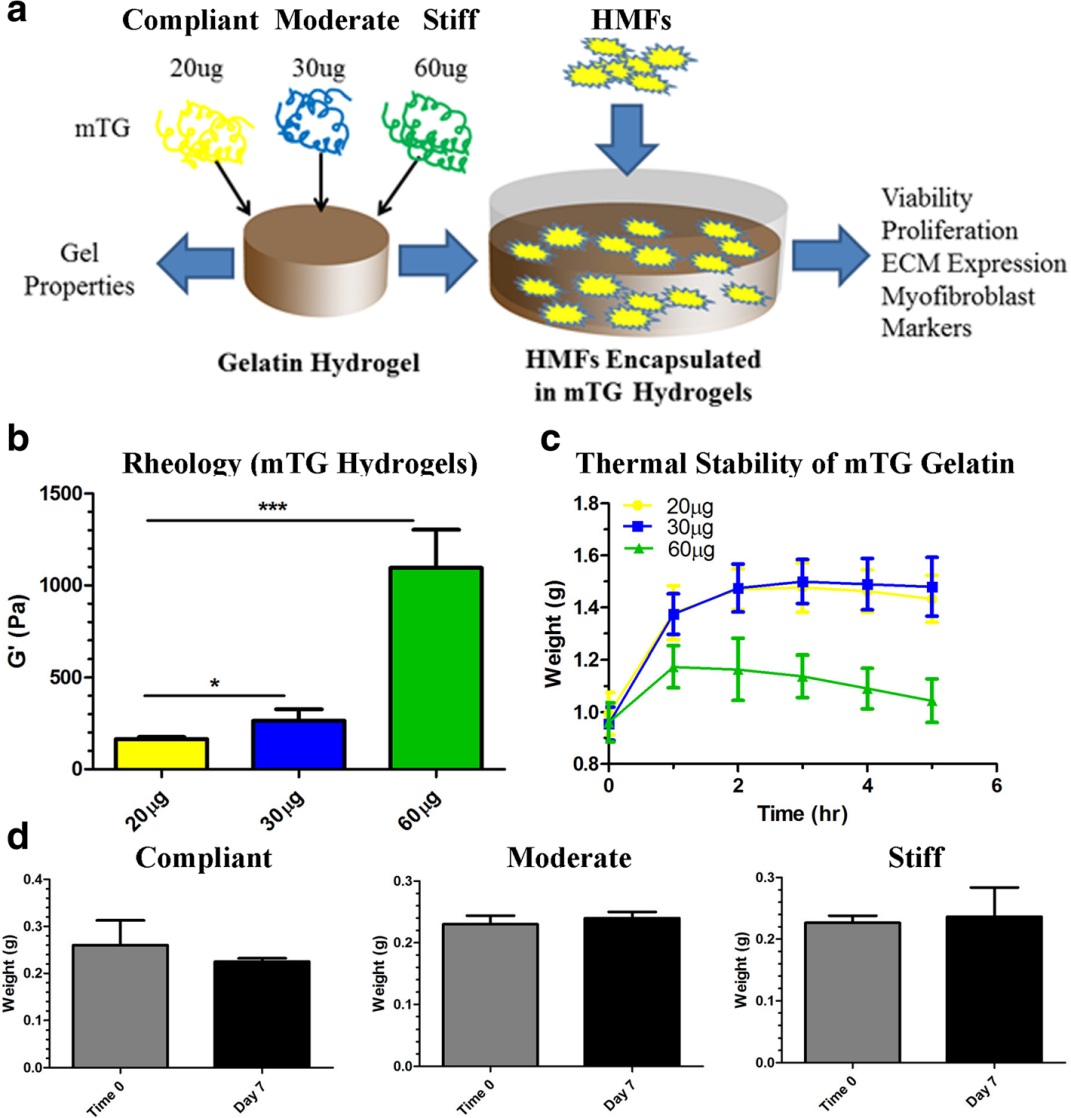
Hydrogel properties. **a** Schematic of hydrogel setup and downstream assays. **b** Rheology was performed on hydrogels cross-linked with 20, 30 and 60 μg/ml mTG. As mTG concentration increased, the elastic modulus (G’) of the hydrogels corresponding increased. The most significant change in G’ is shown for hydrogels cross-linked with 60 μg/ml mTG. **c** Hydrogels cross-linked with 20, 30 and 60 μg/ml mTG were examined over a 5 h period for changes in hydrogel weight following incubation with PBS. Hydrogels cross-linked with 20 and 30 μg/ml mTG exhibited increases in hydrogel weight during the culture period indicative that the gels had swollen during this time frame. Hydrogels cross-linked with 60 μg/ml mTG exhibited a slight, but not significant decrease in hydrogel weight during the incubation period. **d** Differences in the dry hydrogel weight were examined for HMF encapsulated hydrogels at the start of the experiment (time 0) and at the end of the experiment (day 7). Hydrogel weights were similar between time 0 and day 7 measurements for each of the mTG hydrogels. ^*^ *p* ≤ 0.05; ^**^ *p* ≤ 0.01; ^***^ *p* ≤ 0.001

To determine the mechanical stiffness of mTG cross-linked gelatin hydrogels, rheology was used to test the elastic moduli (G’) of hydrogels treated with 20 (compliant), 30 (moderate) and 60 μg/ml (stiff) of mTG. An overview of the methodology and downstream assays used on HMF encapsulated 3D hydrogels is shown in Fig. 2a. Prior to testing the elastic moduli, mTG cross-linked hydrogels were allowed to polymerize for 35–45 min at 37 °C. Following polymerization, the hydrogels were incubated in 1X PBS at 37 °C overnight to mimic cell culture conditions. The following day, PBS was removed from the hydrogels and the elastic moduli were recorded from duplicate samples for compliant, moderate and stiff hydrogels. G’ values corresponded to 200 Pa (20 μg/ml mTG), 300 Pa (30 μg/ml mTG) and 1100 Pa (60 μg/ml mTG) (Fig. 2b). These results demonstrate that with increasing concentrations of mTG, the elastic moduli of the gelatin hydrogels correspondingly increases with the most dramatic change observed for 60 μg/ml of mTG.

### Thermal stability of mTG hydrogels

Next, the thermal stability of mTG cross-linked hydrogels was measured. This factor is important as the hydrogels will need to support long-term growth of HMFs. Furthermore, hydrogel degradation not only alters the mechanical properties of the gel, but may additionally alter its biocompatibility, presumably as a result of cytotoxic breakdown products. After crosslinking the hydrogels with 20, 30 and 60 μg/mL of mTG, the initial hydrogel weight was recorded for triplicate samples. Subsequently, 1 mL of pre-warmed 1X PBS was added to each of the hydrogels and the hydrogels were placed in an incubator at 37 °C. At each hour for a total of 5 h, the weight of the mTG hydrogels was measured following careful removal of the PBS. The average weight of the compliant and moderate hydrogels increased between 1 and 2 h after which time, the weights remained relatively stable (Fig. 2c). A slight decrease in weight was observed for the moderate hydrogel between 4 and 5 h of incubation (Fig. 2c). Contrary to results from compliant and moderate hydrogels, the average weight for the stiff hydrogels increased between 0 and 1 h, but steadily decreased at each subsequent time point analyzed (Fig. 2c). These results indicate that while some loss of hydrogel weight occurred during the analysis period, there were no significant changes in the weight of these.

Finally, to determine if the hydrogel weight changed during the culture period as a result of cell-mediated degradation, we measured the dry weight of HMF encapsulated hydrogels at the beginning of culture following a 1.5-2 h period of hydrogel swelling (time 0) and at the end of the culture period (day 7). No significant changes in hydrogel weight were recorded for each of the mTG conditions (Fig. 2d), suggesting that the hydrogels maintain their bulk integrity throughout the culture period.

### HMF morphology in 3D mTG hydrogels

**Fig. 3:**
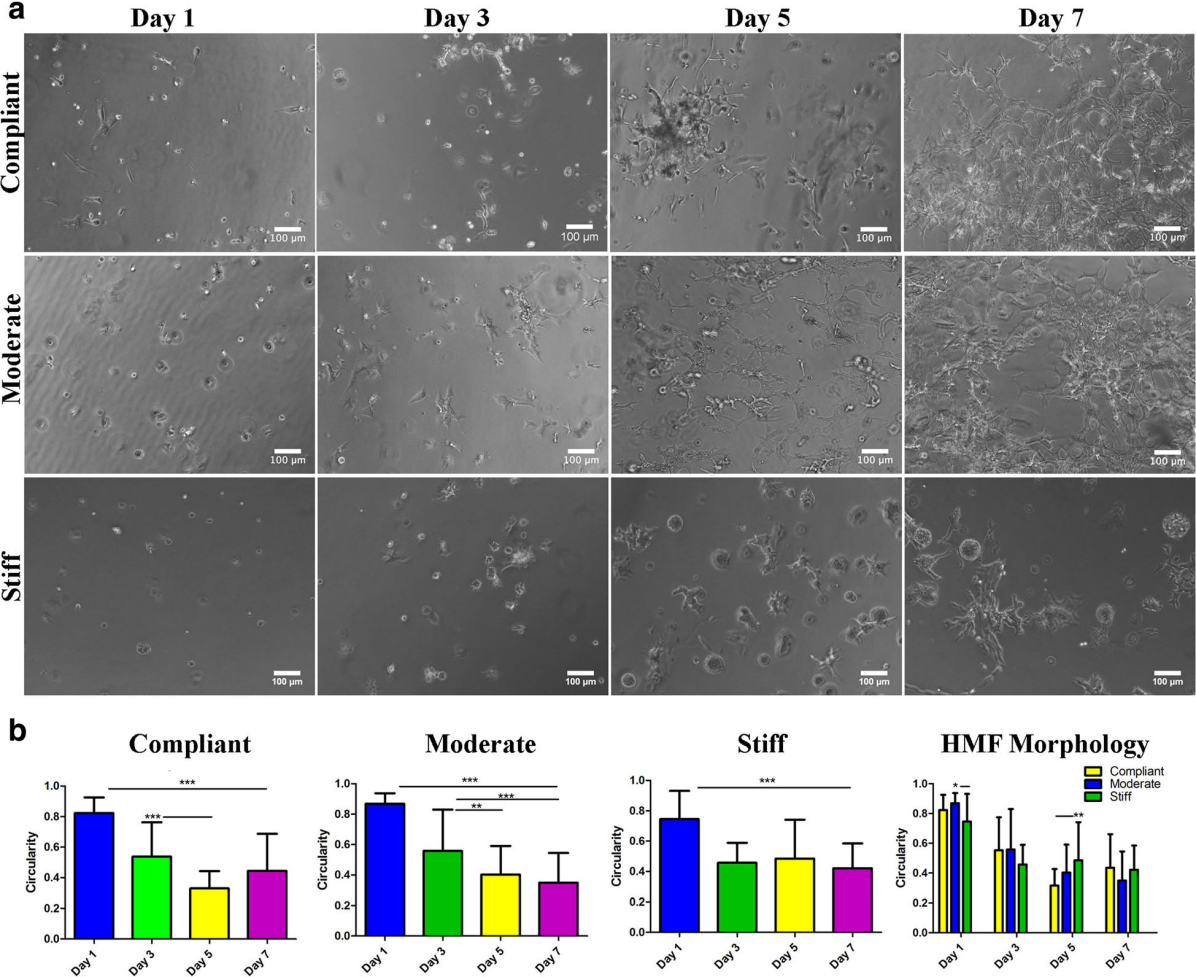
HMF morphology in mTG hydrogels. **a** HMFs encapsulated in compliant, moderate and stiff hydrogels were examined for differences in cell morphology during the culture period. At day 1, HMFs in all tested mTG concentrations appeared rounded and started to spread, forming elongated spindle-like morphologies at day 3. At day 5, HMFs in compliant and moderate hydrogels assumed a more spindle-like morphology while HMFs in stiff hydrogels appeared to have a larger cell body with thicker cellular protrusions. After 7 days in culture, HMFs in compliant and moderate hydrogels retained their spindle-like morphology from day 5, but occupied a greater area of the hydrogel. HMFs in stiff mTG hydrogels still exhibited a large cell body with multiple large cellular protrusions at day 7, an observation similar to that for day 5. **b** HMF morphology was quantified based on the circularity where 0 indicates an elongated structures and 1 indicates a perfect circle. Overall, HMFs became more elongated during the culture period in each of the mTG hydrogels. For stiff hydrogels, HMF morphology didn’t change much after day 3 of culture. Comparing hydrogel conditions, HMFs were more elongated in stiff hydrogels at days 1 and 3, compliant hydrogels at day 5 and moderate hydrogels at day 7. ^*^ *p* ≤ 0.05; ^**^ *p* ≤ 0.01; ^***^ *p* ≤ 0.001

To determine whether increasing 3D mechanical stiffness altered the morphology of HMFs, HMFs encapsulated in compliant, moderate and stiff hydrogels were evaluated at days 1, 3, 5, and 7 of culture. On day 1, HMFs within compliant hydrogels appeared more spindle-like and exhibited a greater degree of spreading than HMFs in the moderate and stiff hydrogels (Fig. 3a). By day 3, HMFs in all hydrogel conditions took on multiple morphologies such as rounded with and without small projections (stiff hydrogels) as well as spindle-like with some spreading (compliant and moderate hydrogels) (Fig. 3a). HMFs in stiff hydrogels also appeared larger in size than HMFs in the compliant and moderate hydrogels at day 3 (Fig. 3a). At day 5, HMFs in the stiff hydrogels had markedly larger cell bodies than HMFs in compliant and moderate hydrogels which appeared elongated and spindle-shaped (Fig. 3a). It was on the last day of culture, day 7, that HMFs in the compliant and moderate hydrogels became confluent and continued to exhibit an elongated, spindle-like morphology (Fig. 3a). Contrary, HMFs in the stiff hydrogels exhibited a large, rounded morphology with large cellular protrusions, but weren’t nearly as confluent as HMFs in the compliant and moderate hydrogels (Fig. 3a). To quantify differences in cellular morphology, ImageJ was used to measure circularity where 0 indicates a perfect circle and values approaching 1 indicate an elongated structure. Using this tool, it was found that HMFs gradually assumed an elongated morphology during the culture period although these changes weren’t as evident for stiff hydrogels where patterns of HMFs elongation stayed relatively consistent between days 3-7 (Fig. 3b). Comparing hydrogel conditions, we found that HMFs in stiff hydrogels were more elongated at days 1 and 3, HMFs in compliant hydrogels at day 5 and HMFs in moderate hydrogels at day 7. Together, these results indicate that HMFs exhibited a more spindle-like morphology in compliant and moderate hydrogels. In stiff hydrogels, HMFs exhibited a more rounded morphology with numerous cellular protrusions. Furthermore, HMFs assumed a more elongated morphology during the culture period in each of the hydrogel conditions with the exception of HMFs in stiff hydrogels.

**Fig. 4:**
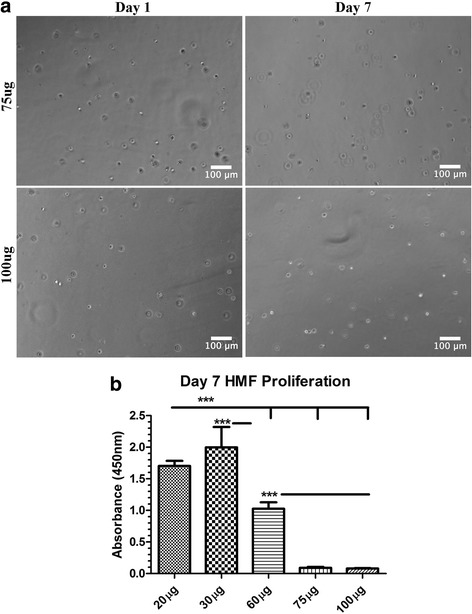
HMF morphology and proliferation in 75 and 100 μg/ml mTG hydrogels. **a** HMFs were encapsulated in 75 and 100 μg/ml mTG hydrogels and allowed to incubate for 7 days. HMFs displayed a rounded morphology at days 1 and 7 in culture. **b** HMFs encapsulated in mTG cross-linked hydrogels were examined for changes in proliferation at day 7 of culture. HMFs in 75 and 100 μg/ml mTG hydrogels exhibited a significant decrease in proliferation in comparison to HMFs in compliant, moderate and stiff hydrogels. HMFs in the moderate hydrogels exhibited the most significant change in proliferation. ^*^ *p* ≤ 0.05; ^**^ *p* ≤ 0.01; ^***^ *p* ≤ 0.001

To determine whether gelatin hydrogels cross-linked with higher concentrations of mTG supported cell growth, HMFs were encapsulated in 7.5% gelatin hydrogels treated with 75 and 100 μg of mTG. The morphology of the HMFs didn’t change over the 7 day culture period and appeared rounded throughout (Fig. 4a). In comparison to HMFs encapsulated in compliant, moderate and stiff hydrogels, HMF proliferation was significantly reduced in 75 and 100 μg hydrogels at day 7 of culture, suggesting that high concentrations of mTG do not support growth of 3D encapsulated HMFs (Fig. 4b).

### HMF viability and proliferation in mTG hydrogels

**Fig. 5:**
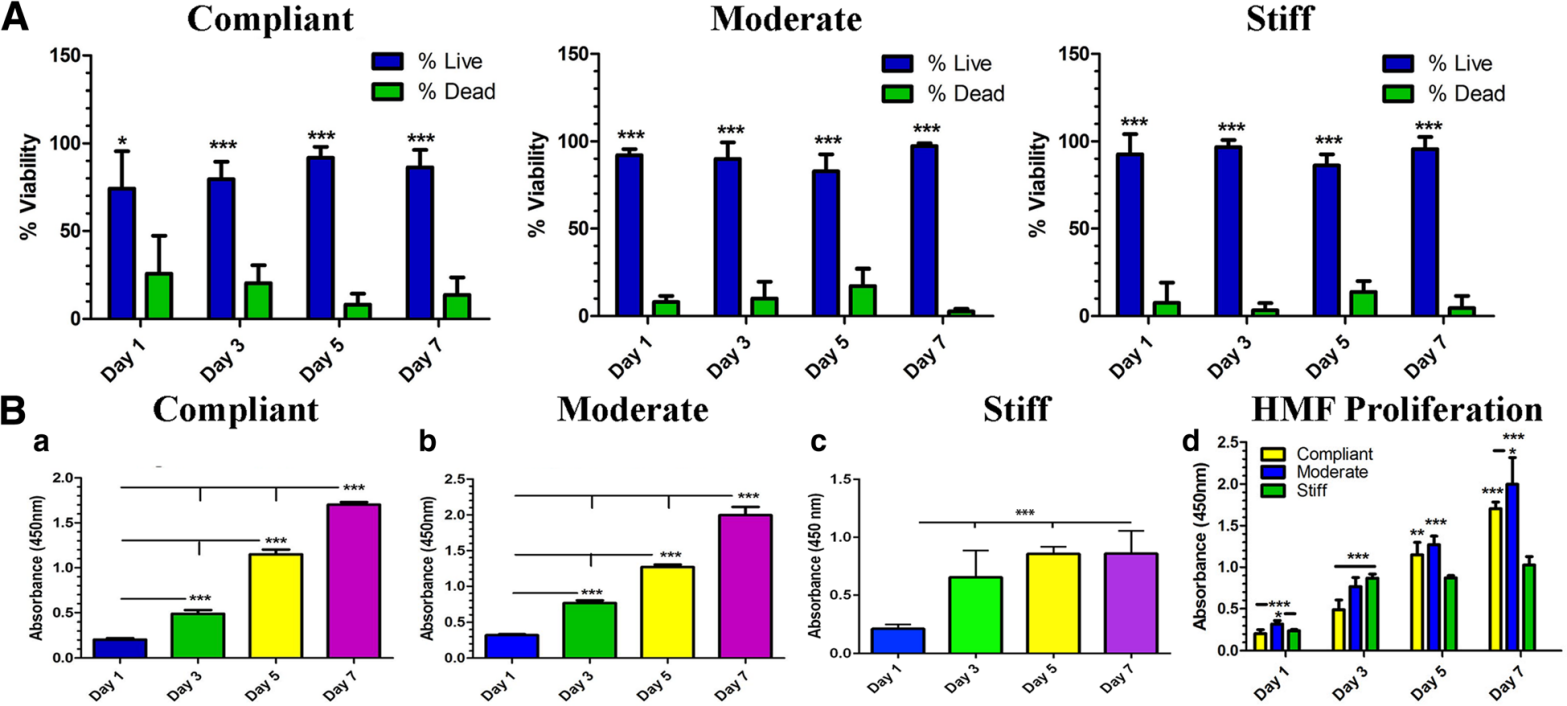
HMF viability and proliferation encapsulated in mTG hydrogels. HMFs encapsulated in compliant, moderate and stiff hydrogels were assessed for changes in viability and proliferation during the culture period. **a** Overall, HMFs exhibited significantly greater numbers of live versus dead cells in all hydrogel conditions and at all time points tested. **ba,b** During the culture period, the rate of HMF proliferation in compliant and moderate hydrogels significantly increased. **bc** The rate of HMF proliferation in stiff hydrogels increased between days 1 and 5, with no appreciable changes between days 5 and 7. **bd** Overall, HMFs encapsulated in moderate hydrogels exhibited the most significant changes in proliferation at all time points tested with the exception of day 3. ^*^ *p* ≤ 0.05; ^**^ *p* ≤ 0.01; ^***^ *p* ≤ 0.001

To test viability during the culture period, HMFs encapsulated in compliant, moderate and stiff hydrogels were incubated in a calcein/ethidium bromide solution and assessed for changes in the number of live/dead cells using fluorescence microscopy. All analyses were conducted at days 1, 3, 5, and 7 of the culture period. As shown in Fig. 5, HMFs exhibited a significantly greater percentage of viable cells in comparison to dead cells in all tested hydrogel conditions and at each time point evaluated. These results indicate that HMFs are viable in 3D hydrogels cross-linked with up to 60 μg/ml of mTG. Representative images from live/dead HMFs for each tested time point and in each of the hydrogel conditions are shown in Additional file 1: Figure S1, Additional file 2: Figure S2 and Additional file 3: Figure S3.

Changes in proliferation of HMFs encapsulated in compliant, moderate and stiff hydrogels was additionally evaluated using the WST assay. Similar to viability analyses, all assessments of HMF proliferation were conducted at days 1, 3, 5 and 7 of the culture period. For HMFs encapsulated in compliant and moderate hydrogels, proliferation steadily and significantly increased during the culture period (Fig. 5b a, b). HMF proliferation in stiff hydrogels was significantly increased between days 1 and 3 but plateaued between days 5 and 7 of culture (Fig. 5b c). When comparing proliferation rates for HMFs encapsulated in all hydrogels, it was found that proliferation was generally greater for HMFs encapsulated within the moderate hydrogels in comparison to those encapsulated within the compliant and stiff hydrogels (Fig. 5b d). At each time point assessed, with the exception of day 3, HMF proliferation in the stiff hydrogels was found to be significantly less than that for HMFs encapsulated in the compliant and moderate hydrogels (Fig. 5b d). Combined, these results suggest that HMFs remain viable and proliferate during the 7 day culture period in each of the tested hydrogel conditions. Additionally, these results show that HMFs encapsulated in the stiff hydrogels exhibit an overall lesser rate of proliferation in comparison to HMFs encapsulated in the compliant and moderate hydrogels.

### Myofibroblast expression and function in HMFs encapsulated in mTG hydrogels

**Fig. 6:**
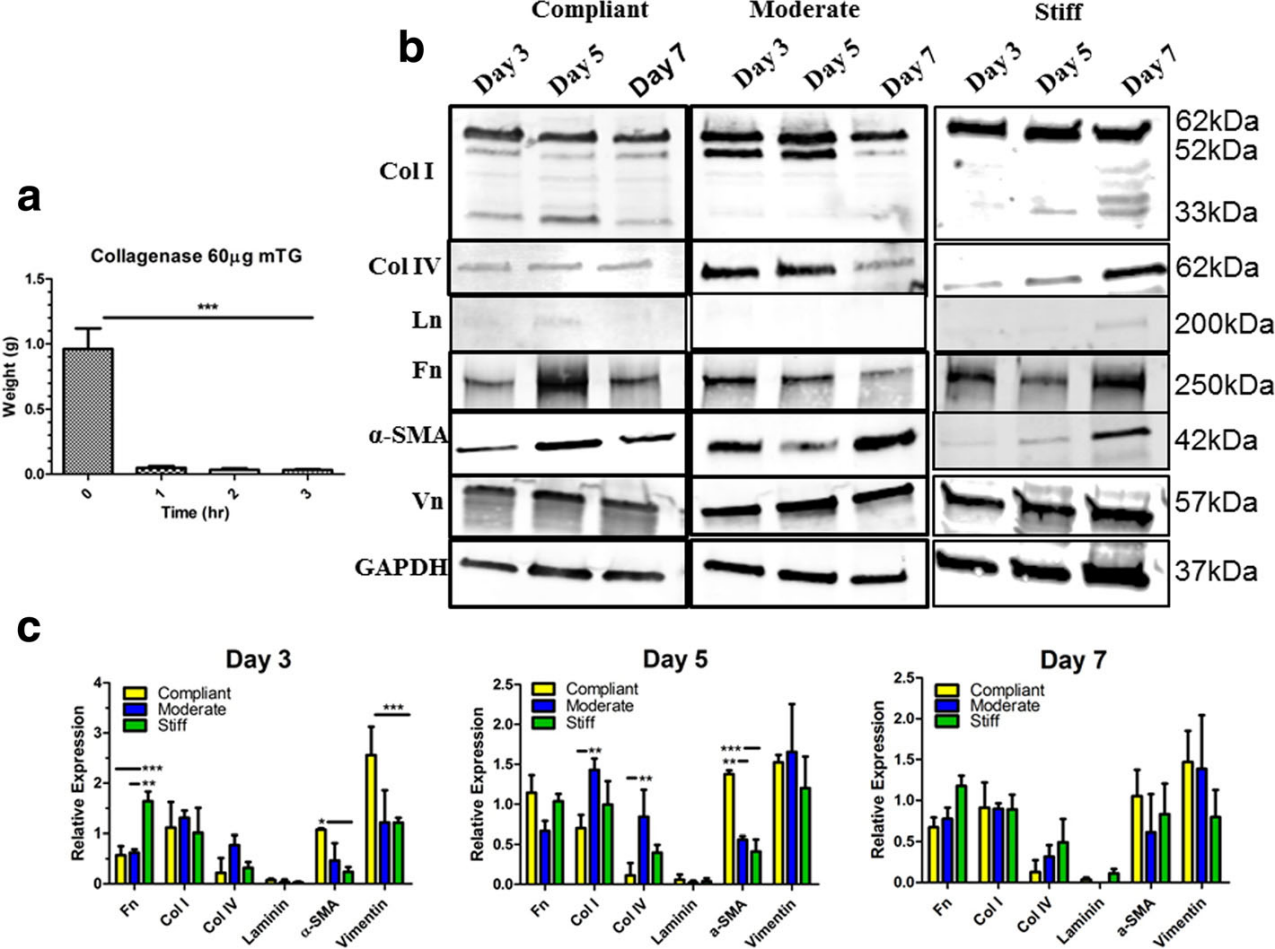
HMF expression of myofibroblast markers and ECM proteins in mTG hydrogels HMFs were isolated from mTG cross-linked hydrogels and analyzed for expression of myofibroblast markers and ECM proteins. **a** Collagenase was used to isolate HMFs from stiff (e.g. 60 μg/ml) mTG hydrogels. Changes in the weight of the hydrogel were monitored during the incubation period wherein 1 h was shown to be a sufficient length of time for dissolution of the hydrogel. **b** Protein lysates from compliant, moderate and stiff hydrogels were subjected to western blot for analysis of myofibroblast and ECM proteins. α-SMA was most highly expressed in compliant hydrogels at day 5 and for moderate and stiff hydrogels at day 7. Collagen I and vimentin expression were upregulated in HMF in moderate and stiff hydrogels in comparison to HMFs in compliant hydrogels. Collagen IV was more abundant in HMFs encapsulated in the moderate and stiff hydrogels with expression greatest at days 3 and 5 for moderate and day 7 for stiff hydrogels. Fibronectin expression was highest overall at days 5 and 7 for HMFs in compliant and stiff hydrogels, respectively. Laminin was detectable, albeit negligibly, at days 5 and 7 for HMFs in compliant and stiff hydrogels, respectively. **c** Fibronectin expression was increased for stiff hydrogels at days 3 (significantly) and day 7 for compliant hydrogels. Collagen I was increased for moderate hydrogels at days 3 and 5 (significantly) and was similarly expressed for all hydrogel conditions at day 7. Collagen IV was increased for moderate hydrogels at days 3 and 5 (significantly) and for stiff hydrogels at day 7. Laminin was negligibly expressed. α-SMA was consistently increased for compliant hydrogels at each time point assessed. Vimentin was significantly increased at day 3 for compliant hydrogels

Previous work has shown that markers of myofibroblasts and ECM proteins are up-regulated in response to matrix stiffness [30–33]. Since 60 μg of mTG yielded the stiffest hydrogel, it was expected that myofibroblast and ECM protein expression would be upregulated during the culture period in comparison to HMFs in 20 (compliant) and 30 μg (moderate) mTG hydrogels. As such, in an effort to determine whether matrix stiffness upregulated the expression of myofibroblast markers in mTG cross-linked 3D gelatin hydrogels, western blot was utilized to assess the expression of myofibroblast markers α-SMA and vimentin and ECM proteins collagen I and IV, laminin and fibronectin in HMFs encapsulated in compliant, moderate and stiff hydrogels at days 3, 5 and 7 of culture. These ECM proteins were selected as previous reports have shown that they are upregulated in myofibroblasts [34–37]. Prior to analyzing changes in myofibroblast marker expression, it was necessary to first assess the conditions needed to liberate HMFs from mTG cross-linked hydrogels. To accomplish this, hydrogels with the greatest mechanical stiffness (e.g. 60 μg mTG) were incubated in 0.5 mg/ml of collagenase. Changes in the weight of the hydrogels were monitored over a 3 h period. After 1 h in collagenase, the 60 μg mTG hydrogels were almost completely digested (Fig. 6a). As such, a period of 1 h in 0.5 mg/ml collagenase was used to liberate HMFs from all mTG cross-linked hydrogels.

Following isolation of HMFs from hydrogels, western blot was utilized to analyze markers of myofibroblast and ECM proteins. Collagen I and vimentin appeared to be similarly expressed at all time points tested in each of the hydrogel conditions (Fig. 6b). Quantification of protein expression though revealed that the 62 kDa band from collagen I was significantly increased at days 3 and 5 in moderate hydrogels and was similarly expressed in compliant and stiff hydrogels (Additional file 4: Figure S4). Vimentin expression was higher in compliant hydrogels at day 3 and for stiff hydrogels at days 5 and 7 although these results weren’t significant (Additional file 4: Figure S4). Comparing protein expression between hydrogels, collagen I was similarly expressed at days 3 and 7 in all hydrogels, but was significantly higher for moderate hydrogels at day 5 (Fig. 6c). For vimentin, expression levels were significantly higher in compliant hydrogels at day 3, but were similarly expressed across the hydrogel conditions at days 5 and 7 with a decrease in expression observed for stiff hydrogels at days 5 and 7 (Fig. 6c). Collagen IV didn’t appear to change over the culture period for HMFs in compliant hydrogels, but was observed to be increased at days 3 and 5 for HMFs in moderate hydrogels and at day 7 for HMFs in stiff hydrogels (Fig. 6b and Additional file 4: Figure S4). Comparing hydrogel conditions, collagen IV was greatest at days 3 and 5 for moderate hydrogels and at day 7 for stiff hydrogels (Fig. 6c). Fibronectin expression appeared most abundant at day 5 for compliant hydrogels, days 3 and 5 for moderate hydrogels and days 3 and 7 for stiff hydrogels (Fig. 6b and Additional file 4: Figure S4). Comparing hydrogel conditions, fibronectin was increased at days 3 (significantly) and 7 for stiff hydrogels and was similar, albeit there was a reduction for the moderate hydrogel, at day 5 (Fig. 6c). α-SMA was most highly expressed in compliant hydrogels at day 5 and for moderate and stiff hydrogels at day 7 (Fig. 6b and Additional file 4: Figure S4). Interestingly, α-SMA was consistently upregulated in compliant hydrogels during the culture period with significant changes observed at days 3 and 5 (Fig. 6c). Laminin expression was overall undetectable for HMFs in compliant, moderate and stiff hydrogels albeit faint bands were visible at days 5 and 7 for HMFs in compliant and moderate hydrogels, respectively (Fig. 6b, c and Additional file 4: Figure S4). Table 2 summarizes the protein expression findings analyzed between hydrogels.

**Table 2:** Summary of protein expression results assessed between hydrogel conditions. Day 3: fibronectin expression was significantly higher for HMFs encapsulated in stiff as compared to compliant and moderate hydrogels. Collagens I and IV were more highly expressed for HMFs encapsulated in moderate as compared to compliant and stiff hydrogels. α-SMA and vimentin expression were significantly higher for HMFs encapsulated in compliant as compared to moderate and stiff hydrogels. Laminin expression was similar between the hydrogel conditions. Day 5: fibronectin expression was higher and α-SMA was significantly higher for HMFs encapsulated in compliant as opposed to moderate and stiff hydrogels. Collagens I and IV were significantly higher while vimentin was higher for HMFs encapsulated in moderate hydrogels as compared to compliant and stiff hydrogels. Laminin expression was similar between the hydrogel conditions. Day 7: fibronectin, collagen IV and laminin expression was higher for HMFs encapsulated in stiff hydrogels as compared to compliant and moderate hydrogels. α-SMA and vimentin expression were increased for HMFs encapsulated in compliant as compared to moderate and stiff hydrogels. Collagen I expression was similar between the hydrogel conditions. ^*^ *p* ≤ 0.05; ^**^ *p* ≤ 0.01; ^***^ *p* ≤ 0.001

	Day 3	Day 5	Day 7
Fibronectin	Stiff (*, **)	Compliant	Stiff
Collagen I	Moderate	Moderate (**)	Similar
Collagen IV	Moderate	Moderate (**)	Stiff
Laminin	Similar	Similar	Stiff
α-SMA	Compliant (*)	Compliant (**, ***)	Compliant
Vimentin	Compliant (***)	Moderate	Compliant

Compliant (20 μg), Moderate (30 μg), Stiff (60 μg)

A defining characteristic of myofibroblasts is their contractile nature, a feature which not only enables wound closure [3], but permits the fibroblast’s ability to remodel and contract collagen hydrogels in 3D [29, 38]. Using a gel contraction assay, we encapsulated HMFs in the various mTG hydrogels to determine whether increasing matrix stiffness in 3D promoted a greater degree of hydrogel contraction, a feature which may be attributed to effects of matrix stiffness on a myofibroblast phenotype. During the 7 day culture period no observable changes in hydrogel contraction were found (data not shown), suggesting that HMFs may not have fully differentiated into a myofibroblast phenotype following encapsulation in the tested hydrogel conditions. Interestingly, it appeared that following release of the hydrogels from the dish, the HMFs became gradually more rounded (data not shown), suggesting that the tension exerted by the attachment of the hydrogel to the sides of the dish is an important mediator for supporting the spreading and growth of the fibroblasts.

Taken together, these results indicate that ECM and myofibroblast proteins in HMFs vary in expression during both the culture period and according to the mechanical stiffness of the hydrogel and further suggest that HMFs may not be fully differentiated into a myofibroblast phenotype as they didn’t contract the mTG hydrogels.

### TGF-β expression from HMFs encapsulated in mTG Hydrogels

**Fig. 7:**
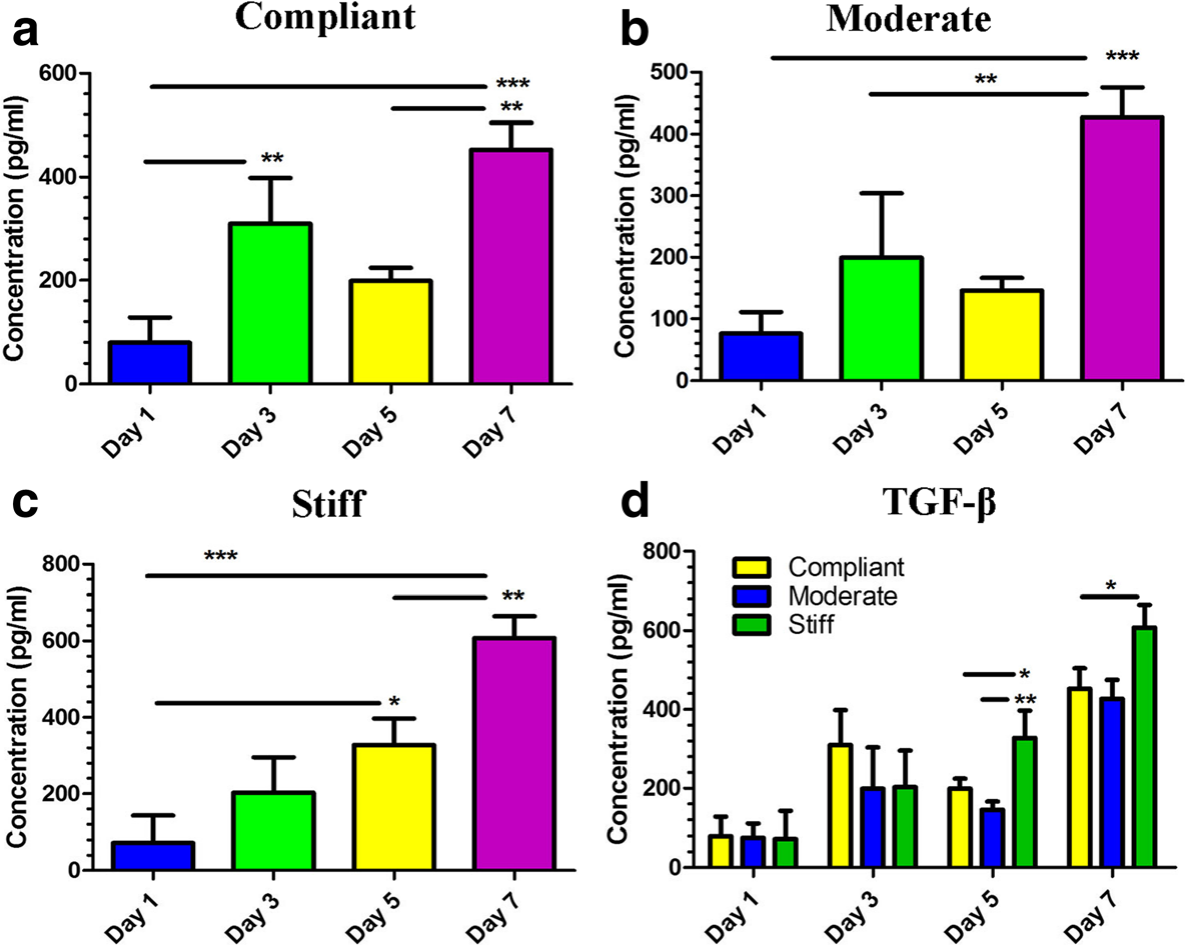
TGF-β production from HMFs encapsulated in mTG hydrogels. HMFs were encapsulated in compliant, moderate and stiff hydrogels and conditioned media was collected at days 1, 3, 5 and 7 of culture for quantification of secreted TGF-β. Overall, TGF-β levels increased during the culture period for HMFs encapsulated in compliant (**a**), moderate (**b**) and stiff (**c**) hydrogels. (**d**) TGF-β production was significantly greater for HMFs encapsulated in stiff hydrogels at days 5 and 7 of culture. ^*^ *p* ≤ 0.05; ^**^ *p* ≤ 0.01; ^***^ *p* ≤ 0.001

TGF-β has been reported to be upregulated following fibroblast transition into myofibroblasts [39]. To determine whether increasing matrix stiffness upregulated TGF-β cytokine levels from 3D cultures of HMFs, conditioned media from HMFs encapsulated in compliant, moderate and stiff hydrogels were evaluated for differences in secreted TGF-β at days 1, 3, 5 and 7 of culture. All data were normalized to TGF-β levels observed in control media (data not shown). Overall, it was found that TGF-β levels significantly increased for HMFs encapsulated in hydrogels at each tested mTG concentration during the culture period (Fig. 7a-c). For HMFs in compliant and moderate hydrogels, there was an increase in TGF-β at day 3 followed by a decrease at day 5 (Fig. 7a-c). When comparing the various mTG hydrogels, HMFs encapsulated in the stiff hydrogels exhibited the most significance difference in TGF-β, a result which wasn’t evident until days 5 and 7 of culture (Fig. 7d). Overall, TGF-β levels weren’t significantly different between HMFs encapsulated in compliant and moderate hydrogels at each tested time point of culture (Fig. 7d). In fact, TGF-β was slightly decreased for HMFs in moderate hydrogels in comparison to HMFs in compliant hydrogels. Together, these results suggest that TGF-β production increases overall during the culture period and is greatest for HMFs encapsulated in stiff hydrogels at days 5 and 7 of culture.

## Discussion

CAFs are a major driver of breast cancer progression, metastasis and therapy resistance [40]. One factor which has been shown to support the transition of fibroblasts into activated myofibroblasts is substrate stiffness [15, 18, 19]. In this paper, we sought to elucidate how mechanical stiffness in a 3D gelatin hydrogel alters the phenotype of HMFs. We show that mTG not only crosslinks the gelatin hydrogels, but that increasing concentrations of mTG produce hydrogels with greater bulk mechanical stiffness as measured using the elastic modulus (G’). Further, these hydrogels support viability and proliferation of encapsulated HMFs. Although myofibroblast-like properties were evident in some of the hydrogel conditions, there wasn’t a clear correlation between substrate stiffness in 3D and transition of HMFs into a myofibroblast-like phenotype, suggesting that it will be important for future experiments to elucidate whether additional factors like biochemical cues or alternate strategies to control matrix stiffness are necessary.

Fibroblasts are spindle shaped, but appear more planar and elongated when grown on a stiff 2D substrate and more rounded when grown on compliant surfaces [41]. Furthermore, stiff substrates facilitate invasive and migratory properties of fibroblasts [42]. As such, we sought to determine whether increasing matrix stiffness promoted a more elongated, spindle-like morphology of 3D encapsulated HMFs. Overall, differences in cell morphology were only apparent between HMFs encapsulated between compliant and moderate hydrogels and with HMFs encapsulated in stiff hydrogels. For instance, HMFs encapsulated in stiff hydrogels had larger cell bodies with shorter and thicker cellular protrusions while HMFs in the compliant and moderate hydrogels exhibited a more elongated, spindle like morphology. These results were largely supported with quantification of circularity in which HMFs assumed a more elongated morphology during the culture period in the compliant and moderate hydrogels. For stiff hydrogels, HMFs became more elongated at day 3; however, few changes in cell morphology were apparent after this time. While it was expected that HMFs in the compliant hydrogel would in general exhibit a more rounded morphology with few cellular extensions, it’s possible that the ability of the cells to remodel the matrix may be more important than the initial substrate rigidity. Given that the substrate stiffness was similar for compliant and moderate hydrogels (200 and 300 Pa, respectively), it would follow that the relative compliance of these hydrogels better supported matrix remodeling and thus cell migration. Contrary, it’s likely that the higher degree of matrix cross-linking in the stiff hydrogels could have impeded the HMFs from fully remodeling the gelatin matrix needed for cell elongation and spreading within the hydrogels. Similar findings were reported by Caliari et al. [43] who found that human mesenchymal stem cells (hMSCs) encapsulated in more cross-linked hyaluronic acid (HA) hydrogels exhibited a more round morphology while hMSCs encapsulated in less cross-linked hydrogels exhibited a more spread morphology, a result the authors attributed to the ability of the hMSCs to remodel the compliant hydrogels. Moreover, a separate report found that the degradability of HA hydrogels influenced the spreading of encapsulated hMSCs [44]. For instance, hydrogels with a higher degree of degradability promoted cell spreading versus those with low degradability [44], further highlighting the importance of cell matrix remodeling as opposed to matrix stiffness effects on cell morphology in 3D. Thus, the ability of cells to remodel their environment, as opposed to increased matrix stiffness, is a likely regulator of cell spreading and morphology in 3D.

During tumorigenesis, the stroma is characterized by an increased number of activated, proliferative fibroblasts [45]. Furthermore, it has been reported that CAFs exhibit a higher proliferative index than normal fibroblasts [46]. These observations prompted us to explore the effect of matrix stiffness on HMF proliferation. It was expected that an increase in matrix stiffness would correlate with an increase in cell proliferation. While we observed statistically significant increases in proliferation over the culture period for HMFs encapsulated in all hydrogels, we found that HMFs encapsulated in the moderate hydrogel exhibited a more significant change in proliferation at almost all tested time points in comparison to HMFs encapsulated in compliant and stiff hydrogels. Interestingly, Munoz-Pinto et al. [47] found that vocal fold fibroblasts exhibited a greater rate of proliferation following encapsulation in PEGDA hydrogels with intermediate molecular weight hyaluronan, a hydrogel with a mechanical stiffness between that of PEGDA hydrogels containing high molecular weight hyaluronan and PEDGA hydrogels alone. Further, this finding may be due to the close relationship between cell proliferation and spreading [48]. Chen et al. [48] demonstrated that endothelial cell spreading and proliferation increased proportionately in response to the geometry of the substrate, suggesting that cell accessibility to matrix cues is important for regulation of cell spreading and growth. In addition, cell-cell contact is also an important regulator of cell proliferation [49]**.** Although cell-cell contact was evident in the stiff hydrogels, it was much less than that observed for HMFs encapsulated in compliant and moderate hydrogels and may be a contributing factor for the observed decreases in proliferation. Despite these explanations for decreased proliferation in the stiff hydrogels, differences in spreading and cell-cell contact cannot explain why HMFs in moderate hydrogels exhibited the highest degree of cell proliferation given that few morphological differences existed between HMFs in compliant and moderate hydrogels. It will be important for future work to further delineate the mechanism(s) responsible for the observed differences in HMF proliferation in mTG cross-linked 3D hydrogels.

One of the most well characterized phenotypic markers indicative of a transition into an activated myofibroblast is the expression of α-SMA [4]. Other markers associated with the myofibroblast phenotype include collagen I [6, 50], fibronectin [6, 37, 51] and vimentin [52] although there is still much debate on the use of vimentin as a myofibroblast marker. To determine whether matrix stiffness resulted in the upregulation of these phenotypic markers along with ECM proteins collagen IV and laminin, we analyzed the expression of these in 3D encapsulated HMFs. Interestingly, α-SMA was most highly expressed for HMFs in compliant hydrogels during the culture period, an observation that was contrary to our expectation. A similar finding was reported by Munoz-Pinto et al. [47] who demonstrated that α-SMA expression in vocal fold fibroblasts didn’t correlate with increasing 3D mechanical stiffness at 21 days of culture. Another recent study also found a reduction in α-SMA mRNA and protein expression in valvular interstitial cells encapsulated in stiff PEG hydrogels [53]. Together with our findings, these reports suggest that an inverse relationship exists between α-SMA expression and matrix stiffness in 3D. Despite this, another study showed that hydrogels containing higher concentrations of collagen promoted greater expression of α-SMA in 3D encapsulated fibroblasts [4]. It’s possible that the higher concentration of collagen in this study increased the availability of cell binding sites in the hydrogels, contributing to the reported increase in α-SMA expression. In our study and that reported by Munoz-Pinto et al. [47], the availability of cell binding sites weren’t altered. In this manner, changes in HMF expression of myofibroblast and ECM proteins should be attributed to the mechanical properties of the hydrogel as opposed to the number of cell binding sites. Despite this, a robust myofibroblast phenotype was not evident in HMFs encapsulated in the stiff hydrogel. The only marker to exhibit upregulation for HMFs in the stiff hydrogels was fibronectin which was increased at days 3 and 7 in comparison to HMFs in compliant and moderate hydrogels. Given these results and those reported by Hinz et al. [4], it will be important for future work to determine whether substrate stiffness and/or increased availability of cell binding sites in 3D drive the myofibroblast phenotype.

TGF-β is a multi-functional cytokine which has been reported to be upregulated following fibroblast transition into myofibroblasts [39] and has also been documented to promote and maintain the myofibroblast phenotype [11]. Therefore, it was hypothesized that if the transition into the myofibroblast phenotype correlated with increased matrix stiffness, then TGF-β production would correspondingly increase with increased matrix stiffness. TGF-β production was found to not only be significantly increased for HMFs encapsulated in all hydrogels at each tested time point during the culture period, but was significantly greater for HMFs encapsulated in stiff hydrogels at day 5 and 7, suggesting that matrix stiffness supports TGF-β production from 3D encapsulated HMFs at later points in culture. These results are in accordance with a prior report demonstrating that cells cultured atop a mechanically stiff hydrogel exhibited greater levels of TGF-β activation, an observation the authors attributed to cell mediated contractility and liberation of matrix-bound TGF-β [54]. Furthermore, TGF-β levels were also reported to be higher in HCT118 cells encapsulated in 25 kPa hydrogels, but declined in gels where the mechanical stiffness was much higher [55]. Overall, these studies support a role for mechanical stiffness in the upregulation of TGF-β.

## Conclusions

In conclusion, we have shown that HMFs are viable and proliferate in gelatin hydrogels crosslinked with various concentrations of mTG and express some myofibroblast markers as matrix stiffness increases. Specifically, we show that cell proliferation in addition to collagens I and IV, vimentin and TGF-β expression were increased in HMFs encapsulated in moderate and stiff hydrogels. On the other hand, α-SMA, considered as an excellent marker for the myofibroblast phenotype, was increased for HMFs in compliant hydrogels during the culture period as compared to HMFs in moderate and stiff hydrogels. Although these studies support a role for matrix stiffness in activating some features of a myofibroblast phenotype, the functional and phenotypic markers indicative of a myofibroblast phenotype weren’t robustly expressed. Another important consideration is that there was very little difference in the mechanical stiffness between the compliant and moderate hydrogel, potentially lending to fewer observed differences between HMFs encapsulated in these hydrogels. Given that the model was suitable for culture of HMFs in hydrogels of ≤1 kPa, this model could conceivably be applied to additional studies in which a relatively soft hydrogel is required. Overall, considering the role that mechanical stiffness has on breast tumor growth, metastasis and therapy resistance [56, 57], features that are in part attributed to the presence of myofibroblasts promoting tissue fibrosis [4], it will be important for future work to determine whether the availability of cell binding sites and/or the mechanical properties of the hydrogel are responsible for driving the myofibroblast phenotype in 3D.

## Additional files

Additional file 1: Figure S1. Live/dead HMFs in compliant hydrogels. Representative images of live cells (GFP) and dead cells (RFP), indicated by arrows, for HMFs encapsulated in compliant hydrogels. (TIFF 2655 kb)

Additional file 2: Figure S2. Live/dead HMFs in moderate hydrogels. Representative images of live cells (GFP) and dead cells (RFP), indicated by arrows, for HMFs encapsulated in moderate hydrogels. (TIFF 3143 kb)

Additional file 3: Figure S3. Live/dead HMFs in stiff hydrogels. Representative images of live cells (GFP) and dead cells (RFP), indicated by arrows, for HMFs encapsulated in stiff hydrogels (TIFF 2954 kb)

Additional file 4: Figure S4. Quantification of protein expression in compliant, moderate and stiff hydrogels. Protein expression for myofibroblast and ECM markers was quantified from encapsulated HMFs and normalized to the GAPDH loading control. Fibronectin expression was significantly higher at day 5 in comparison to days 3 and 7 for HMFs encapsulated in compliant hydrogels. Vimentin expression was markedly higher at day 3 in comparison to days 5 and 7 although the change wasn’t significantly different. For moderate hydrogels, collagens I and IV were higher at days 3 and 5 in comparison to day 7 with significance observed for collagen I. In stiff hydrogels, fibronectin expression was significantly higher at day 3 in comparison to days 5 and 7 and α-SMA was significantly increased at day 7 in comparison to days 3 and 5. While vimentin was upregulated at days 3 and 5 in comparison to day 7, this change wasn’t statistically significant. ^*^ *p* ≤ 0.05; ^**^ *p* ≤ 0.01; ^***^ *p* ≤ 0.001. (TIFF 429 kb)

Additional file 5: Material 1, Circularity 20 μg, Circularity analyses on HMFs encapsulated in 20 μg hydrogels. (XLSX 19 kb)

Additional file 6: Material 2, Circularity 30 μg, Circularity analyses on HMFs encapsulated in 30 μg hydrogels. (XLSX 19 kb)

Additional file 7: Material 3, Circularity 60 μg, Circularity analyses on HMFs encapsulated in 60 μg hydrogels. (XLSX 19 kb)

Additional file 8: Material 4, Cell Viability 20 and 30 μg, Cell viability analyses for HMFs encapsulated in 20 and 30 μg hydrogels. (XLSX 10 kb)

Additional file 9: Material 5, Cell Viability 60 μg, Cell viability analyses for HMFs encapsulated 60 μg hydrogels. (XLSX 12 kb)

Additional file 10: Material 6, Cell Viability 2D mTG, Cell viability analyses for HMFs grown on 2D culture flasks and treated with various concentrations of mTG. (XLSX 14 kb)

Additional file 11: Material 7, ELISA 20, 30 and 60 μg, ELISA for TGF-β for HMFs encapsulated in 20, 30 and 60 μg hydrogels. (XLSX 19 kb)

Additional file 12: Material 8, ELISA Day 3 20, 30 and 60 μg, ELISA for TGF-β for HMFs encapsulated in 20, 30 and 60 μg hydrogels. HMFs encapsulation for 3 days prior to collection of supernatant for TGF-β. (XLSX 29 kb)

Additional file 13: Material 9, Proliferation 75 and 100 μg, Proliferation analyses for HMFs encapsulated in 75 and 100 μg hydrogels. (XLSX 12 kb)

Additional file 14: Material 10, Collagenase 60 μg, Gel weight following collagenase digestion of 60 μg hydrogels. (XLSX 9 kb)

Additional file 15: Material 11, Gel Weight Measurements, Gel weight of HMFs encapsulated in 20, 30 and 60 μg hydrogels. Weights were taken shortly after polymerization and the end of the culture period. (XLSX 13 kb)

Additional file 16: Material 12, Proliferation 20, 30 and 60 μg, Proliferation analyses for HMFs encapsulated in 20, 30 and 60 μg hydrogels. (XLSX 9 kb)

Additional file 17: Material 13, Rheology 20, 30 and 60 μg, Rheology measurements for HMFs encapsulated in 20, 30 and 60 μg hydrogels. (XLSX 15 kb)

Additional file 18: Material 14, Thermal stability 20, 30 and 60 μg, Analyses of gel encapsulated HMFs for changes in gel weights following culture in a 37C incubator. (XLSX 11 kb)

Additional file 19: Material 15, Western Blot Quantification 20, 30 and 60 μg, Quantification of band intensity for western blots performed on HMFs encapsulated in 20, 30 and 60 μg hydrogels. (XLSX 43 kb)

Additional file 20: Material 16, Western Blot 20, 30 and 60 μg, Western blots performed on HMFs encapsulated in 20, 30 and 60 μg hydrogels. (PPTX 18885 kb)

## Abbreviations

CAFs: Carcinoma associated fibroblasts
ECM: Extracellular matrix
FAP: Fibroblast activation protein
HMFs: Human mammary fibroblasts
MMP: Matrix metalloproteinase
mTG: Microbial transglutaminase
Pa: Pascal
PBS: Phosphate buffered saline
PEG: Poly(ethylene) glycol
SDF1: Stromal derived factor 1
TGF-β: Transforming growth factor beta
α-SMA: Alpha smooth muscle actin
