# Supplementary figures and images for: Mechanically tuned 3 dimensional hydrogels support human mammary fibroblast growth and viability
Source: BMC Cell Biol. 2017 Dec 16;18:35. doi: 10.1186/s12860-017-0151-y (PMC5732527; doi:10.1186/s12860-017-0151-y)

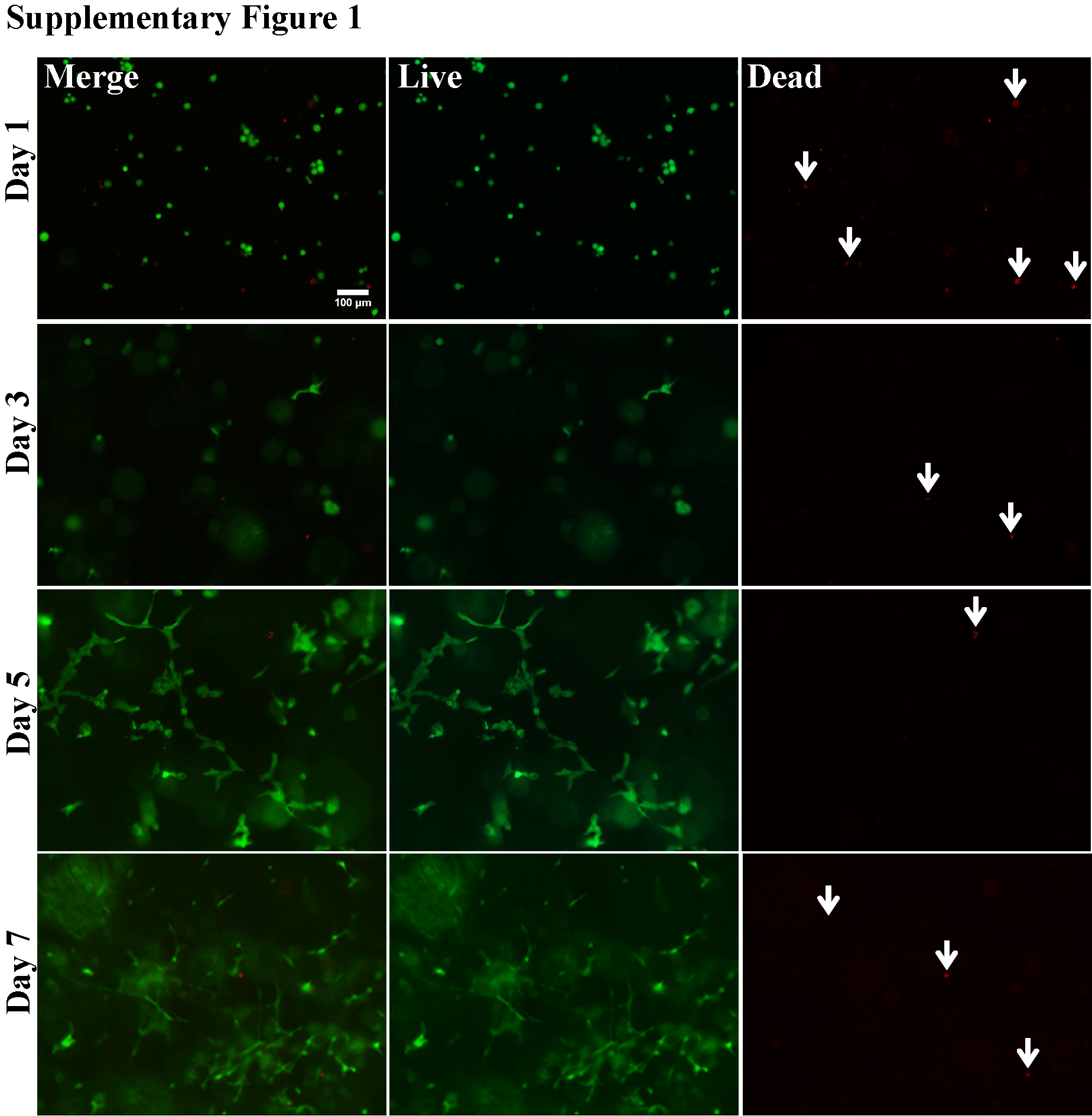

Supplement: Supplementary file 1 — Live/dead HMFs in compliant hydrogels. Representative images of live cells (GFP) and dead cells (RFP), indicated by arrows, for HMFs encapsulated in compliant hydrogels. (TIFF 2655 kb) [file 12860_2017_151_MOESM1_ESM.tif]

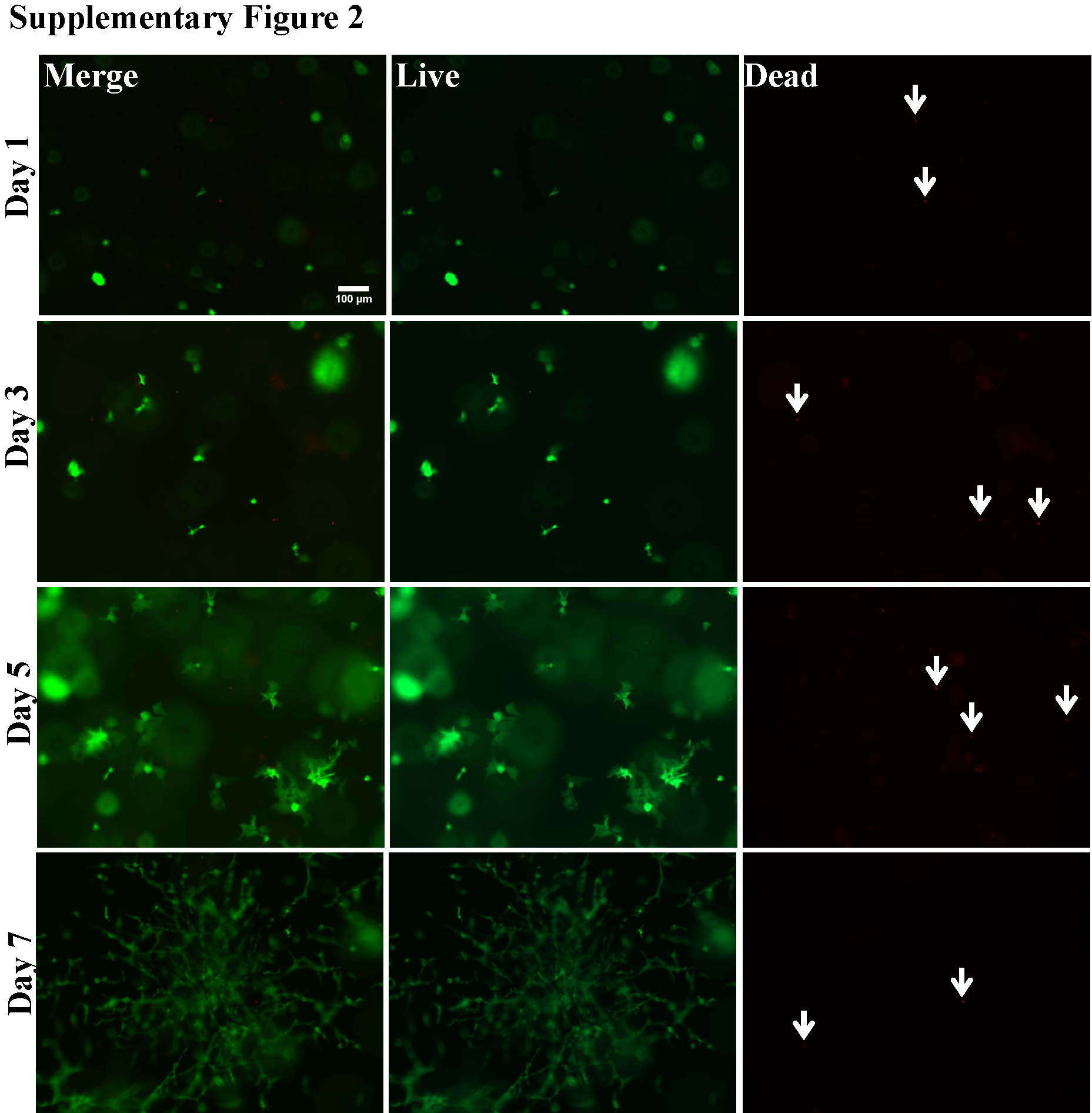

Supplement: Supplementary file 2 — Live/dead HMFs in moderate hydrogels. Representative images of live cells (GFP) and dead cells (RFP), indicated by arrows, for HMFs encapsulated in moderate hydrogels. (TIFF 3143 kb) [file 12860_2017_151_MOESM2_ESM.tif]

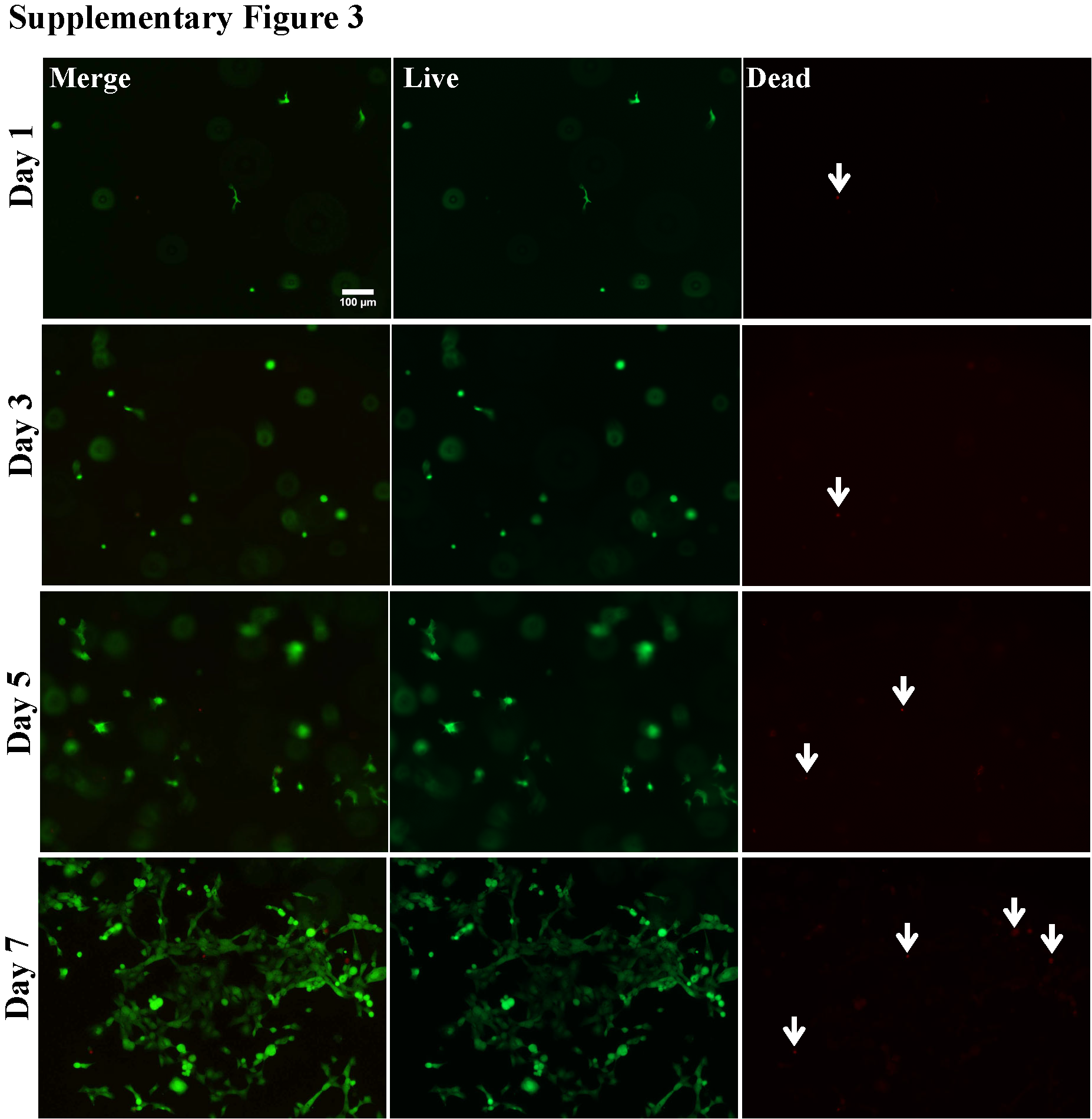

Supplement: Supplementary file 3 — Live/dead HMFs in stiff hydrogels. Representative images of live cells (GFP) and dead cells (RFP), indicated by arrows, for HMFs encapsulated in stiff hydrogels (TIFF 2954 kb) [file 12860_2017_151_MOESM3_ESM.tif]

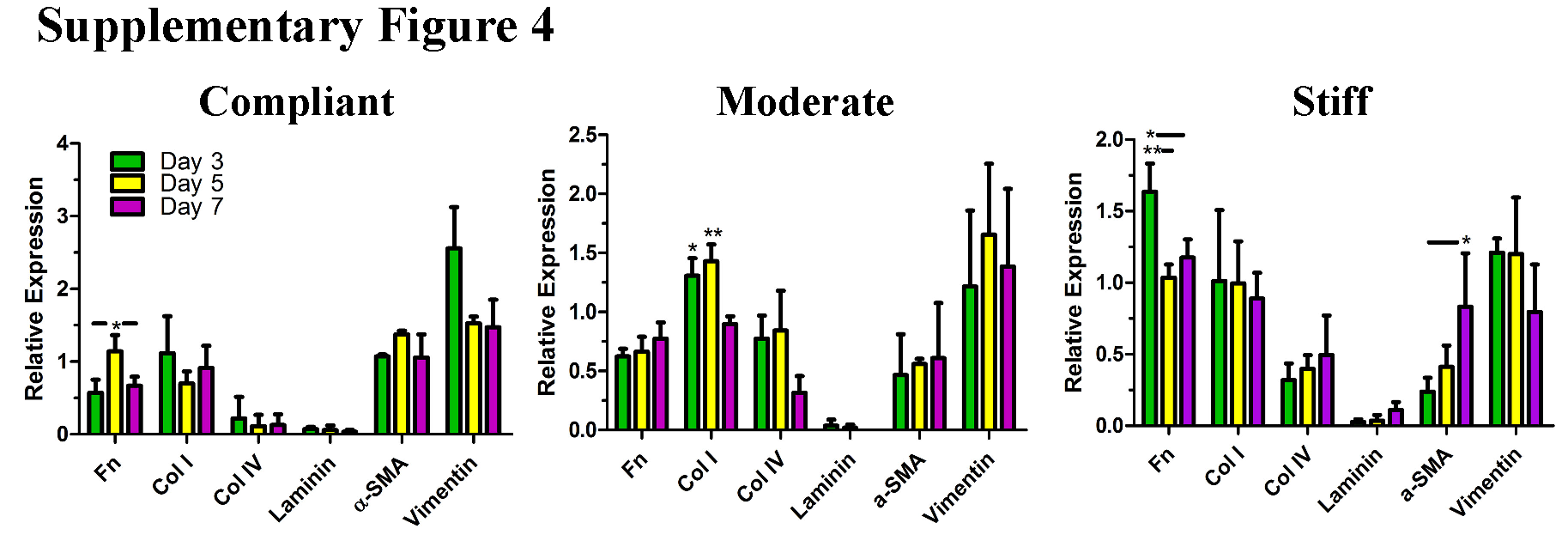

Supplement: Supplementary file 4 — Quantification of protein expression in compliant, moderate and stiff hydrogels. Protein expression for myofibroblast and ECM markers was quantified from encapsulated HMFs and normalized to the GAPDH loading control. Fibronectin expression was significantly higher at day 5 in comparison to days 3 and 7 for HMFs encapsulated in compliant hydrogels. Vimentin expression was markedly higher at day 3 in comparison to days 5 and 7 although the change wasn’t significantly different. For moderate hydrogels, collagens I and IV were higher at days 3 and 5 in comparison to day 7 with significance observed for collagen I. In stiff hydrogels, fibronectin expression was significantly higher at day 3 in comparison to days 5 and 7 and α-SMA was significantly increased at day 7 in comparison to days 3 and 5. While vimentin was upregulated at days 3 and 5 in comparison to day 7, this change wasn’t statistically significant. * p ≤ 0.05; ** p ≤ 0.01; *** p ≤ 0.001. (TIFF 429 kb) [file 12860_2017_151_MOESM4_ESM.tif]
