# Supplementary material for: Mechanically tuned 3 dimensional hydrogels support human mammary fibroblast growth and viability
Source: BMC Cell Biol. 2017 Dec 16;18:35. doi: 10.1186/s12860-017-0151-y (PMC5732527; doi:10.1186/s12860-017-0151-y)
Supplement: Supplementary file 20 — Material 16, Western Blot 20, 30 and 60 μg, Western blots performed on HMFs encapsulated in 20, 30 and 60 μg hydrogels. (PPTX 18885 kb) [file 12860_2017_151_MOESM20_ESM.pptx]

## Slide 1
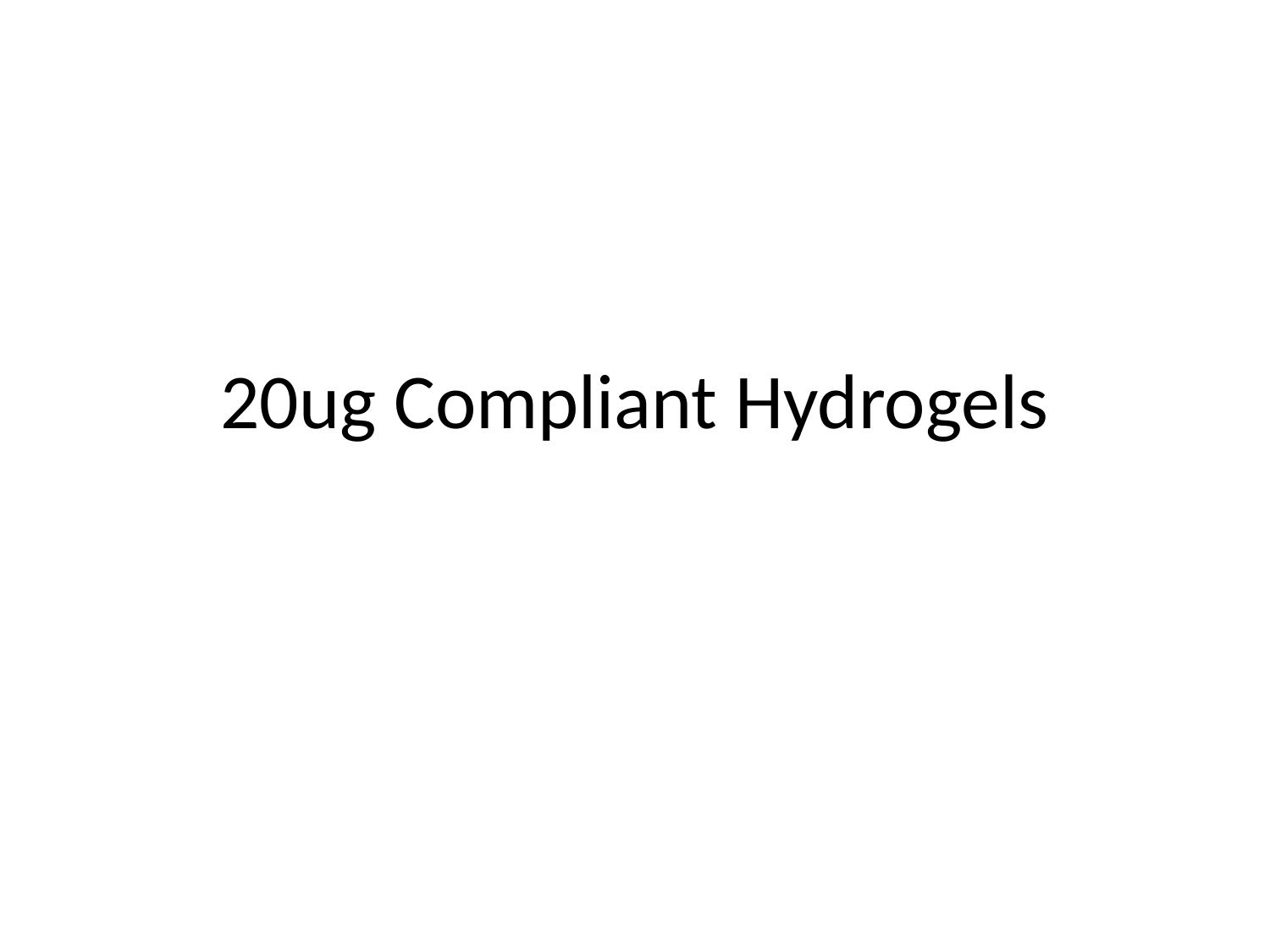

# 20ug Compliant Hydrogels

## Slide 2
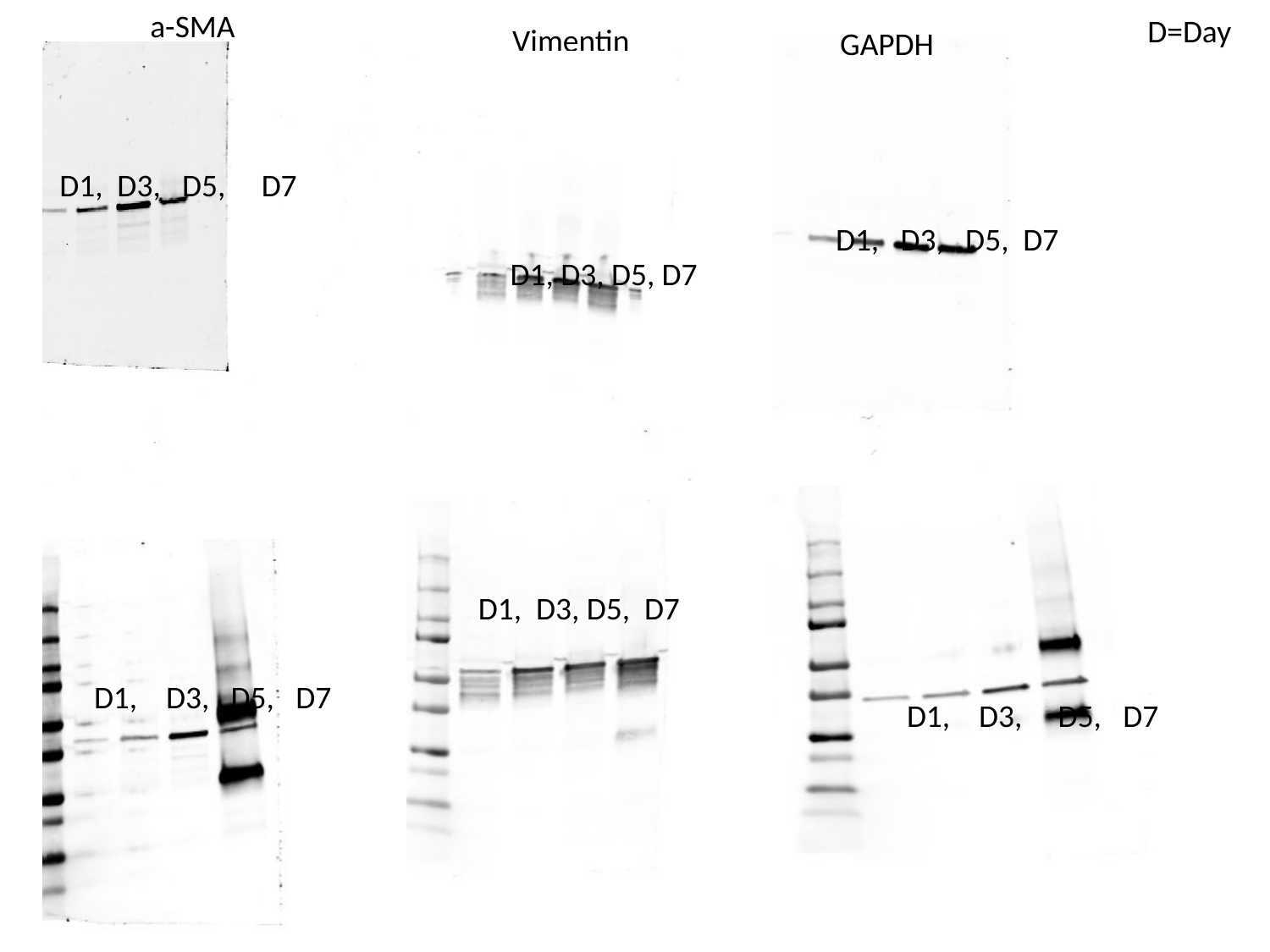

a-SMA
D=Day
 Vimentin
GAPDH
D1, D3, D5, D7
 D1, D3, D5, D7
D1, D3, D5, D7
D1, D3, D5, D7
 D1, D3, D5, D7
D1, D3, D5, D7

## Slide 3
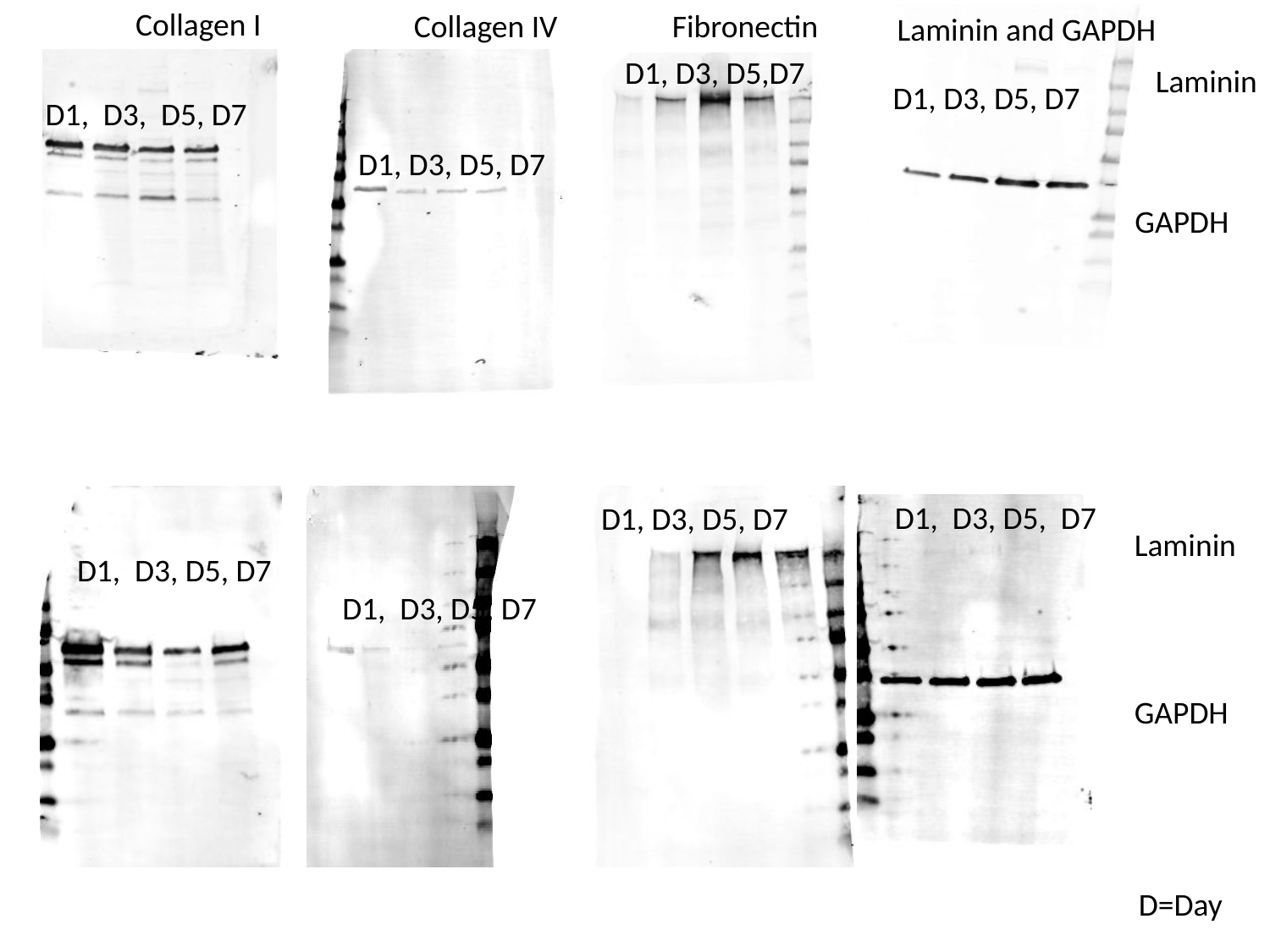

Collagen IV
 Fibronectin
Collagen I
Laminin and GAPDH
 D1, D3, D5,D7
Laminin
 D1, D3, D5, D7
 D1, D3, D5, D7
 D1, D3, D5, D7
GAPDH
 D1, D3, D5, D7
 D1, D3, D5, D7
Laminin
 D1, D3, D5, D7
 D1, D3, D5, D7
GAPDH
D=Day

## Slide 4
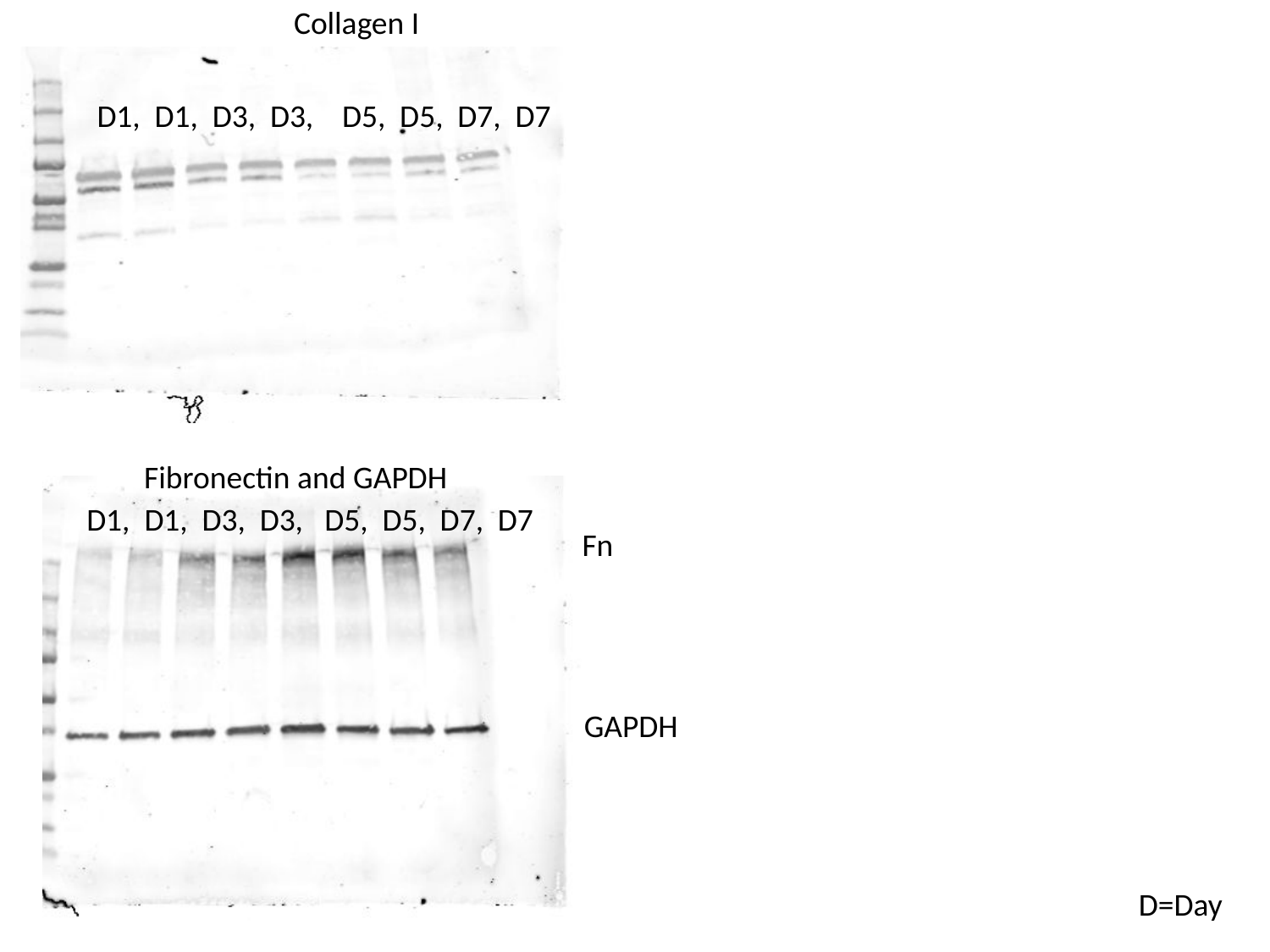

Collagen I
D1, D1, D3, D3, D5, D5, D7, D7
 Fibronectin and GAPDH
D1, D1, D3, D3, D5, D5, D7, D7
Fn
GAPDH
D=Day

## Slide 5
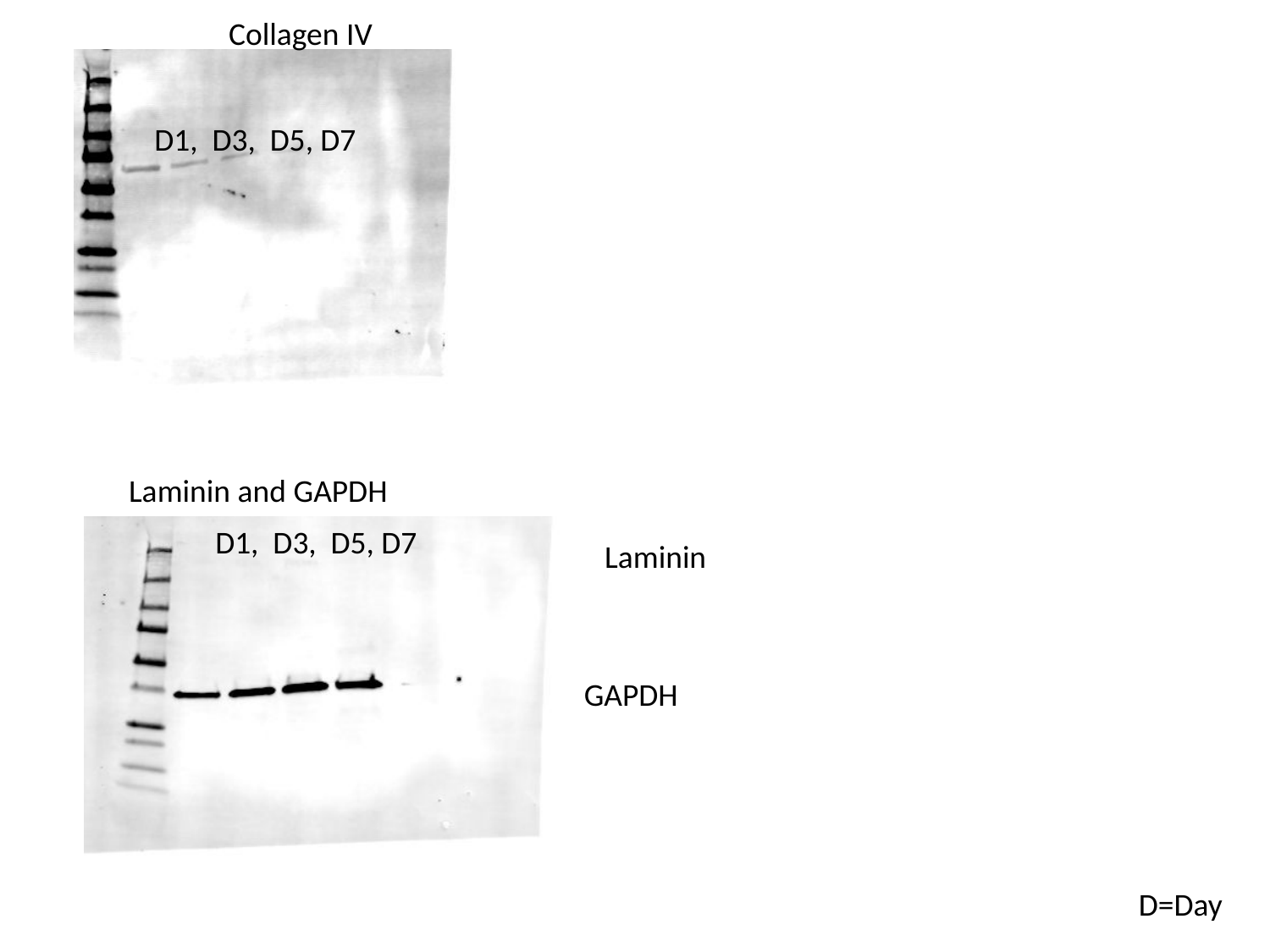

Collagen IV
 D1, D3, D5, D7
Laminin and GAPDH
 D1, D3, D5, D7
Laminin
GAPDH
D=Day

## Slide 6
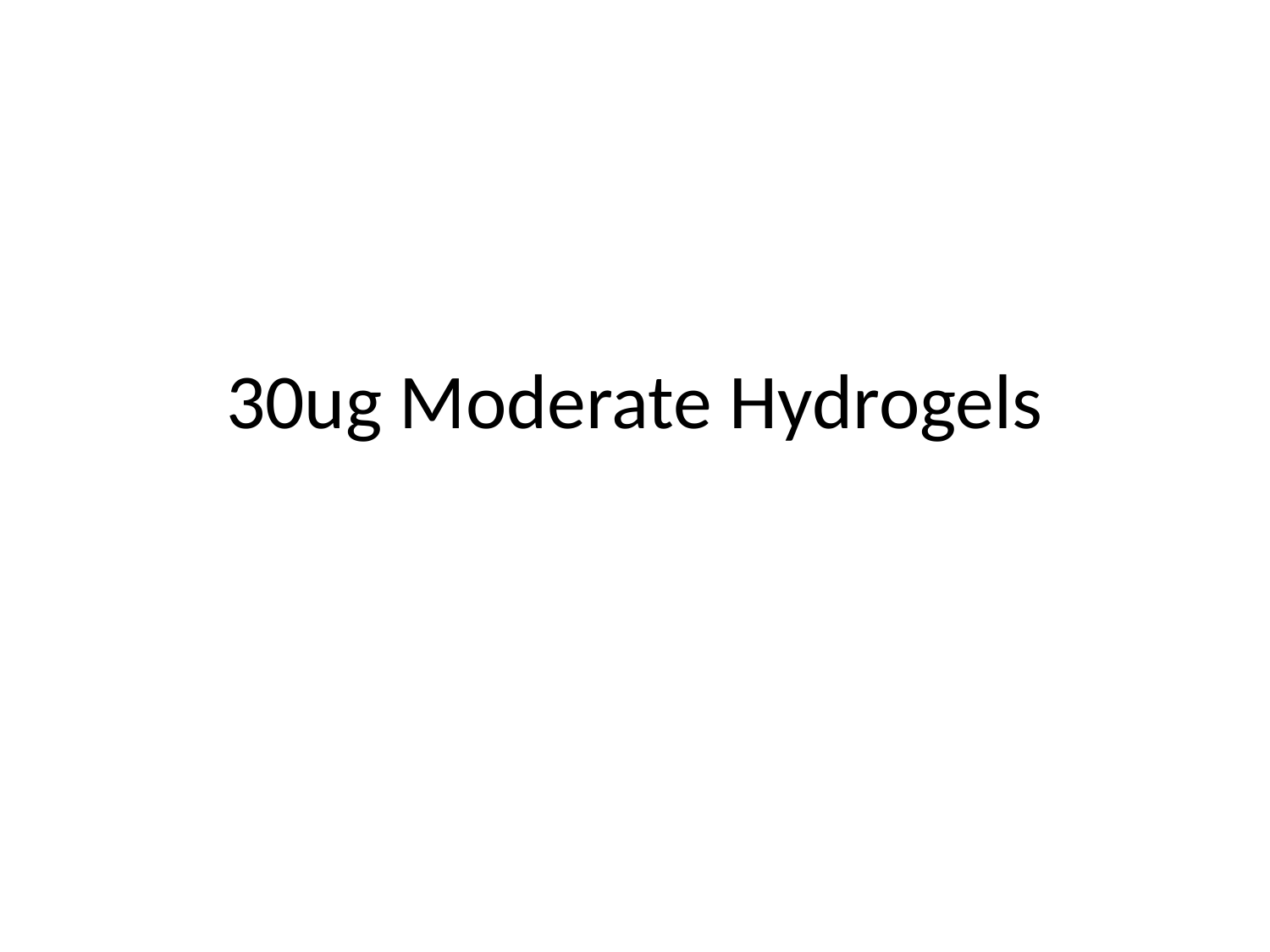

# 30ug Moderate Hydrogels

## Slide 7
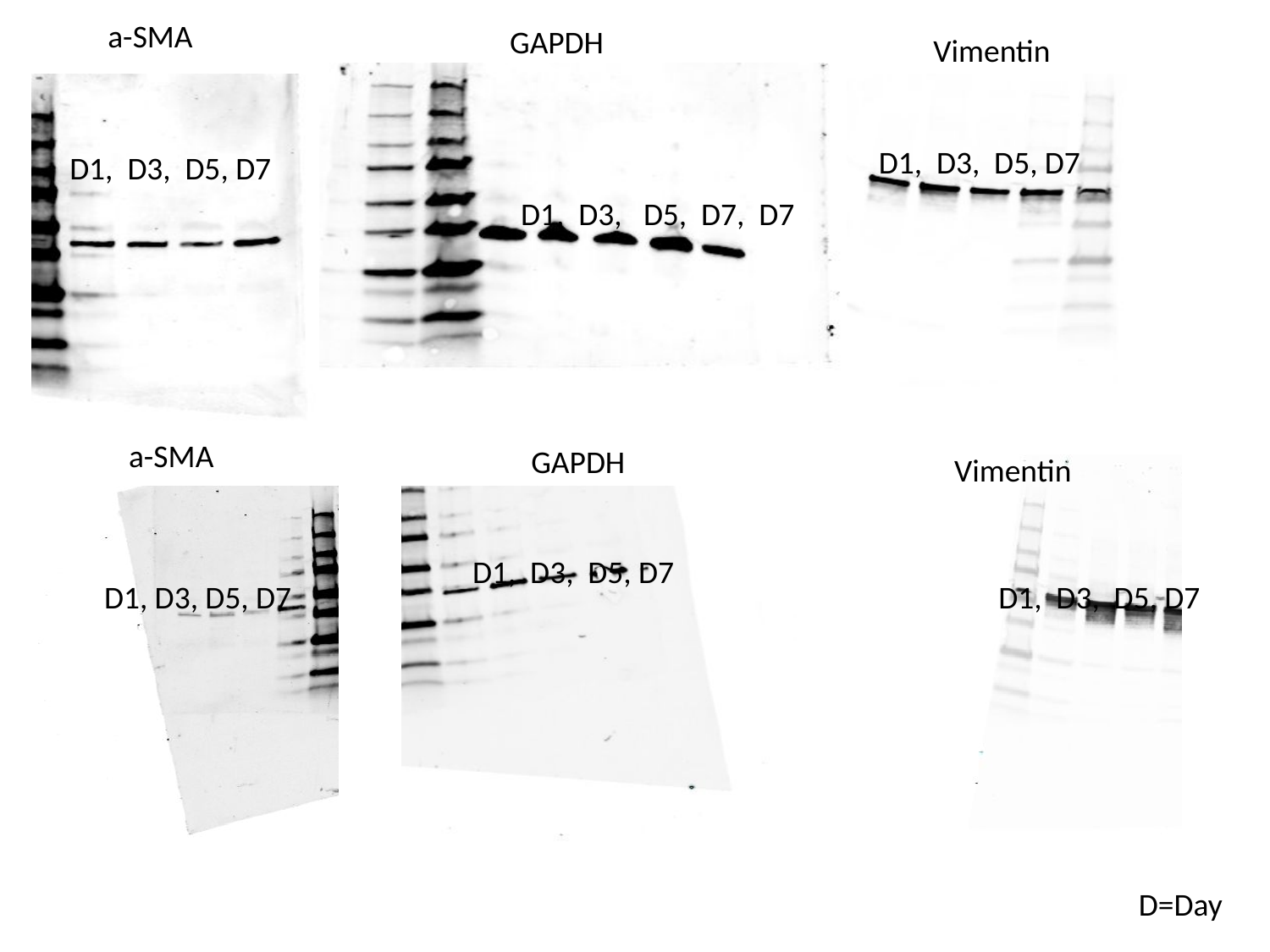

a-SMA
GAPDH
Vimentin
 D1, D3, D5, D7
 D1, D3, D5, D7
 D1, D3, D5, D7, D7
a-SMA
GAPDH
Vimentin
 D1, D3, D5, D7
 D1, D3, D5, D7
 D1, D3, D5, D7
D=Day

## Slide 8
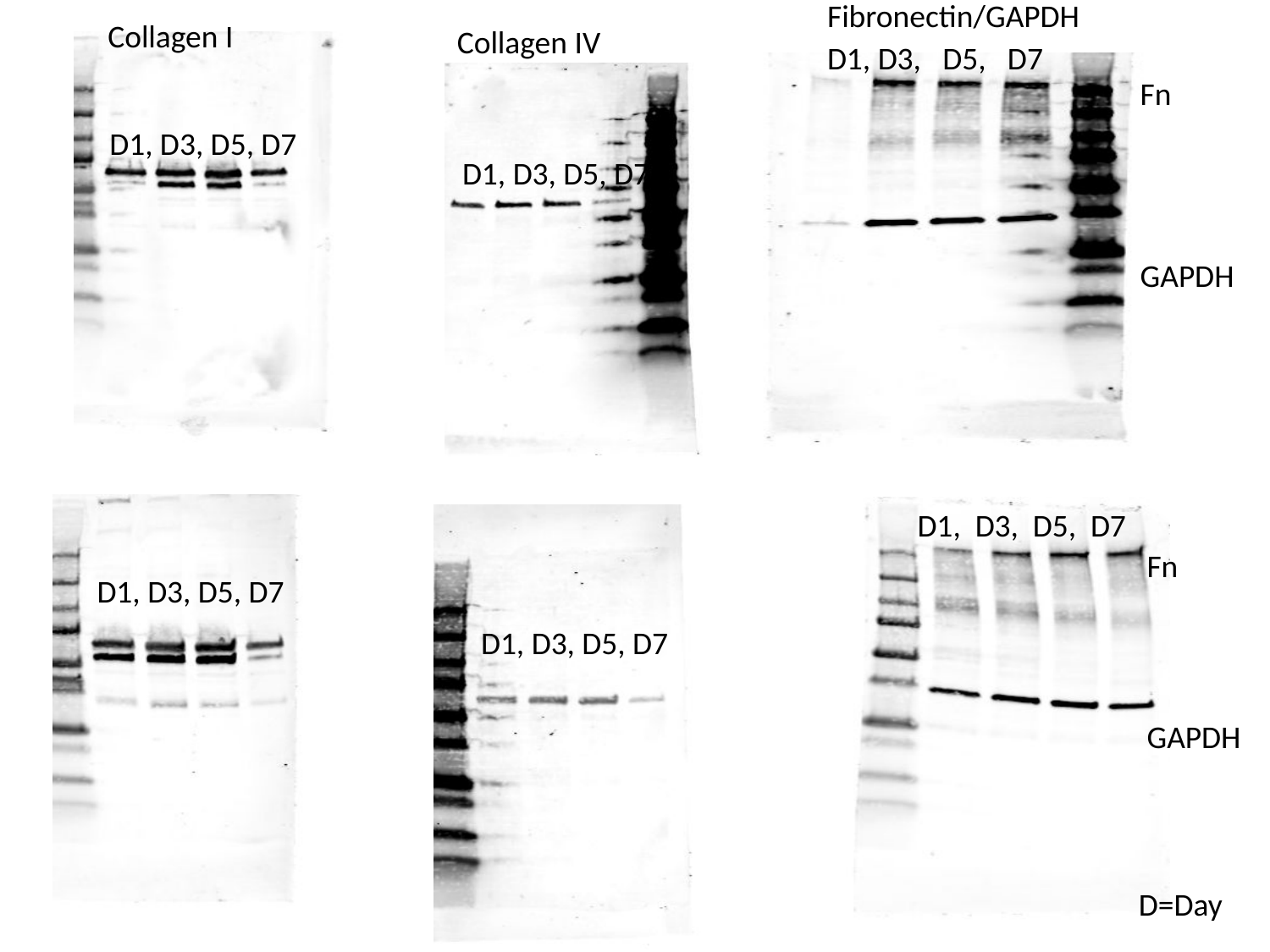

Fibronectin/GAPDH
Collagen I
Collagen IV
D1, D3, D5, D7
Fn
D1, D3, D5, D7
D1, D3, D5, D7
GAPDH
D1, D3, D5, D7
Fn
D1, D3, D5, D7
D1, D3, D5, D7
GAPDH
D=Day

## Slide 9
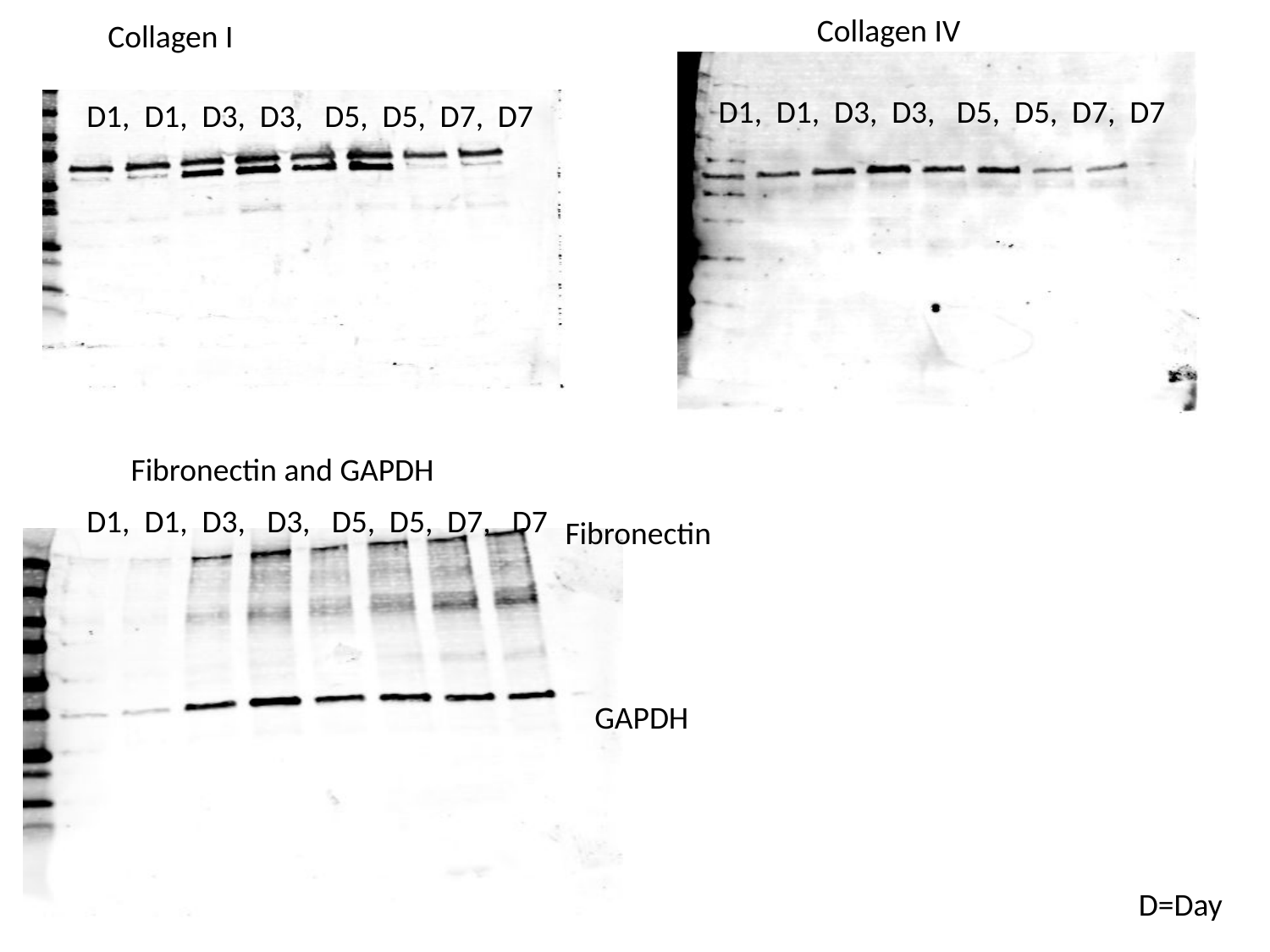

Collagen IV
Collagen I
D1, D1, D3, D3, D5, D5, D7, D7
D1, D1, D3, D3, D5, D5, D7, D7
Fibronectin and GAPDH
D1, D1, D3, D3, D5, D5, D7, D7
Fibronectin
GAPDH
D=Day

## Slide 10
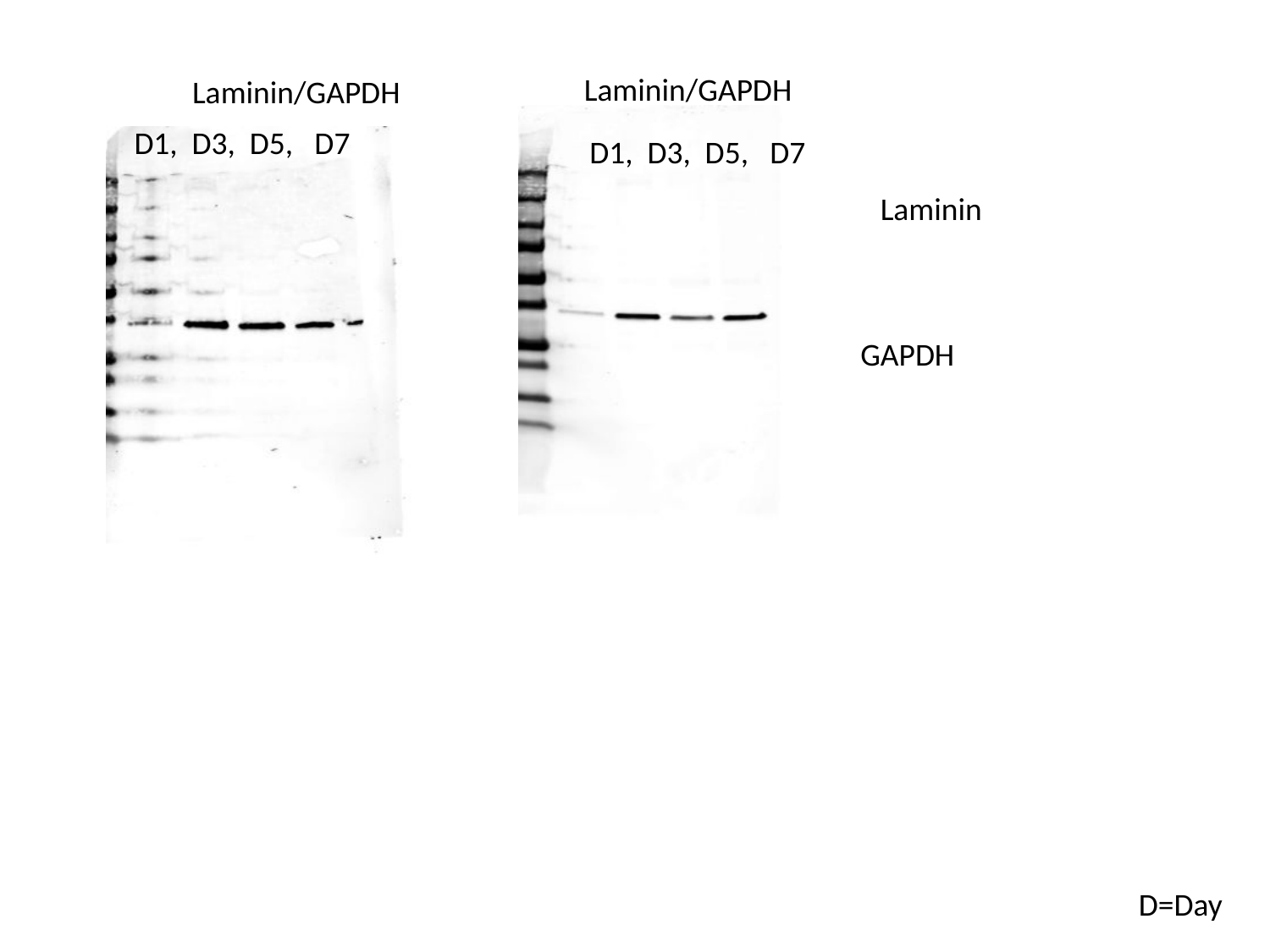

Laminin/GAPDH
Laminin/GAPDH
D1, D3, D5, D7
D1, D3, D5, D7
Laminin
GAPDH
D=Day

## Slide 11
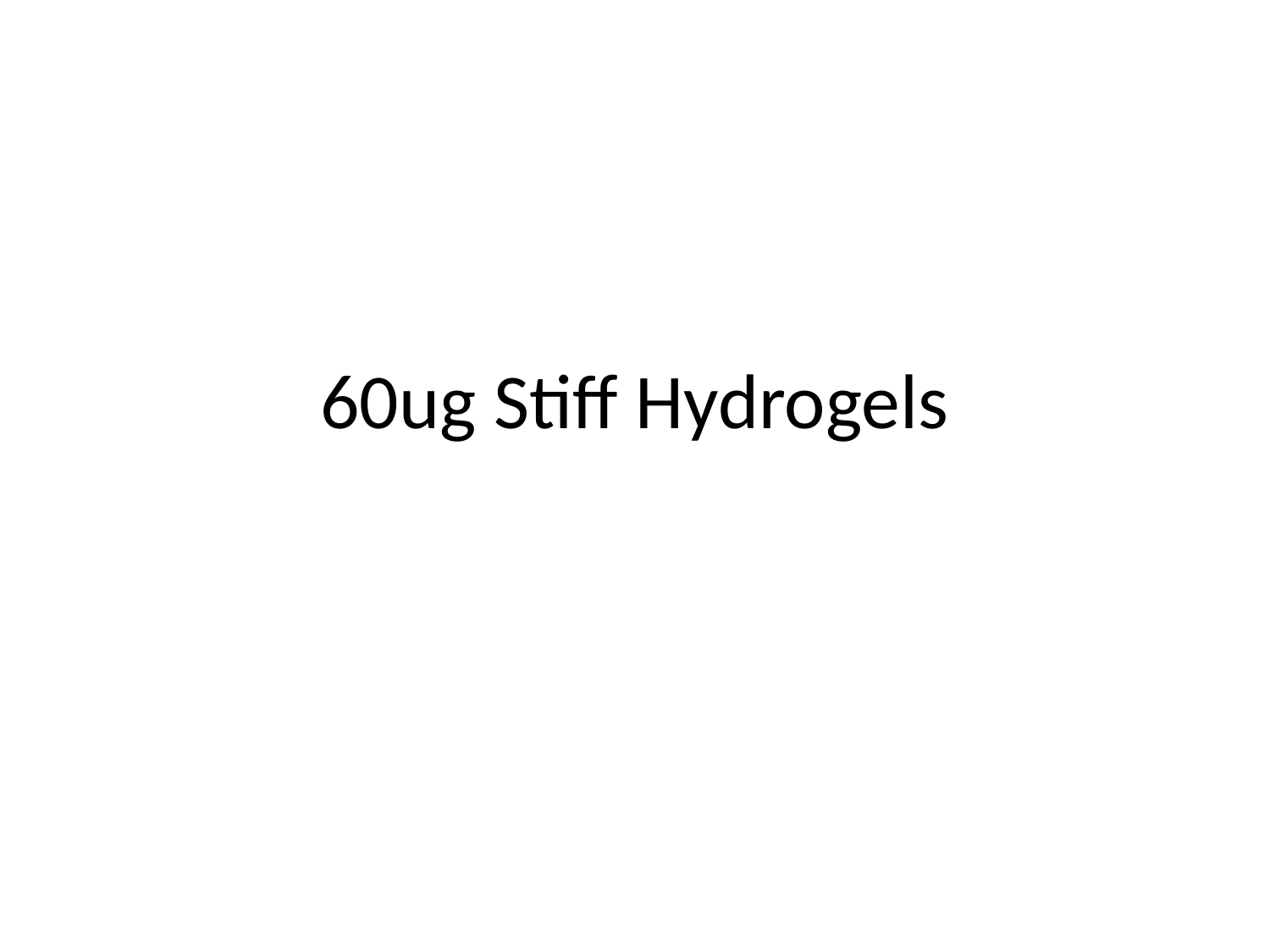

# 60ug Stiff Hydrogels

## Slide 12
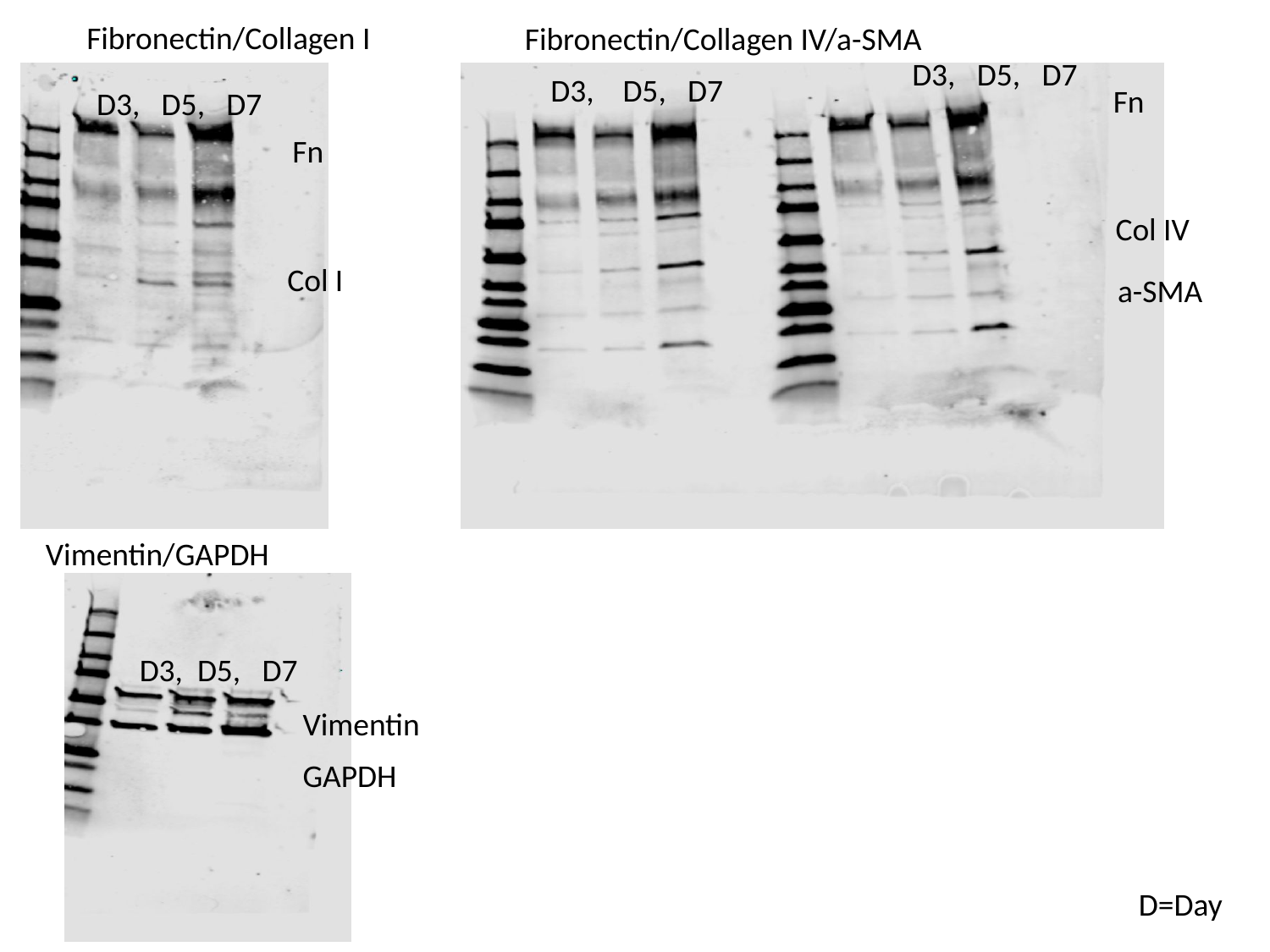

Fibronectin/Collagen I
 Fibronectin/Collagen IV/a-SMA
D3, D5, D7
D3, D5, D7
Fn
D3, D5, D7
Fn
Col IV
Col I
a-SMA
 Vimentin/GAPDH
D3, D5, D7
Vimentin
GAPDH
D=Day

## Slide 13
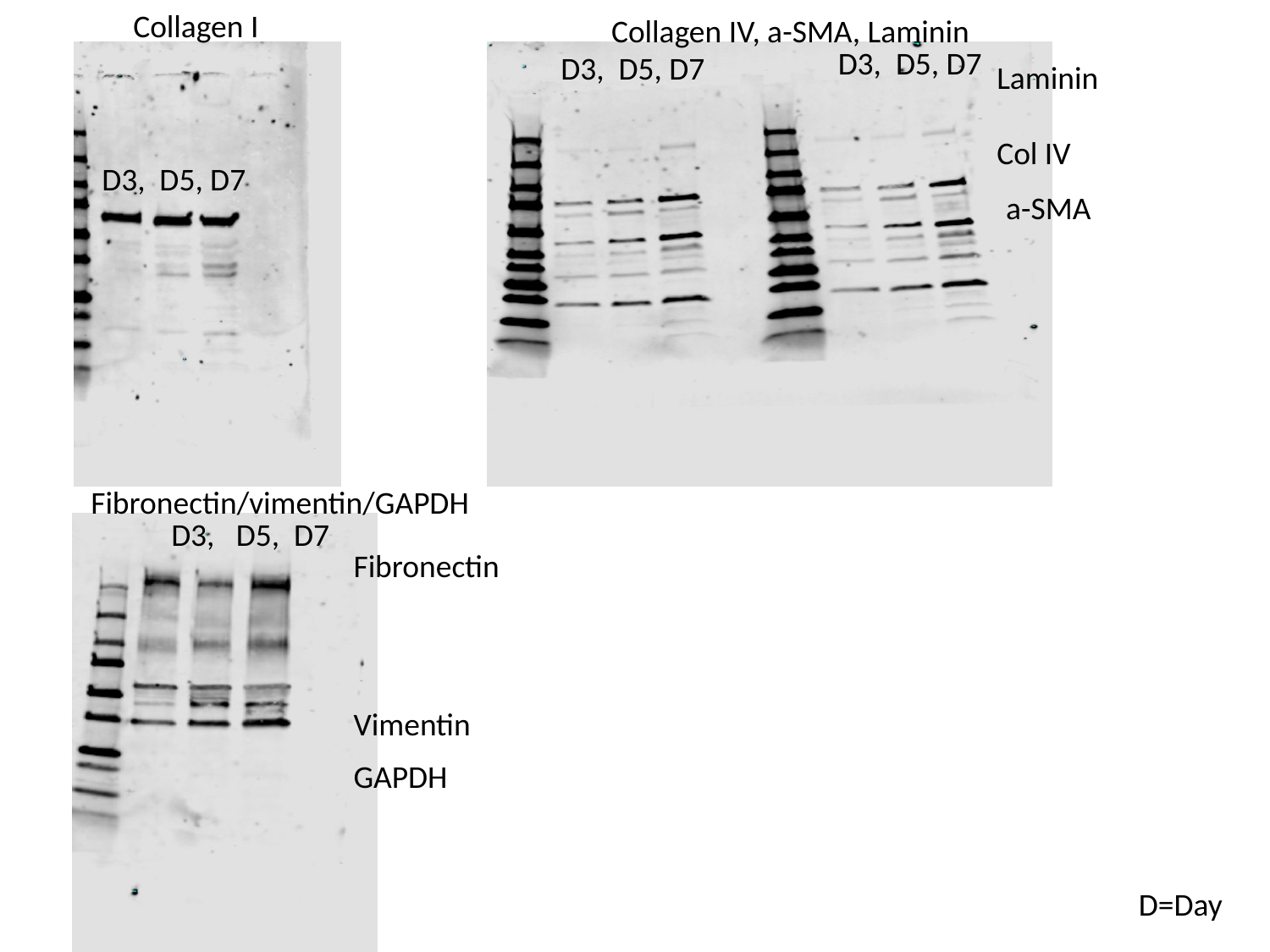

Collagen I
 Collagen IV, a-SMA, Laminin
D3, D5, D7
D3, D5, D7
Laminin
Col IV
D3, D5, D7
a-SMA
 Fibronectin/vimentin/GAPDH
D3, D5, D7
Fibronectin
Vimentin
GAPDH
D=Day

## Slide 14
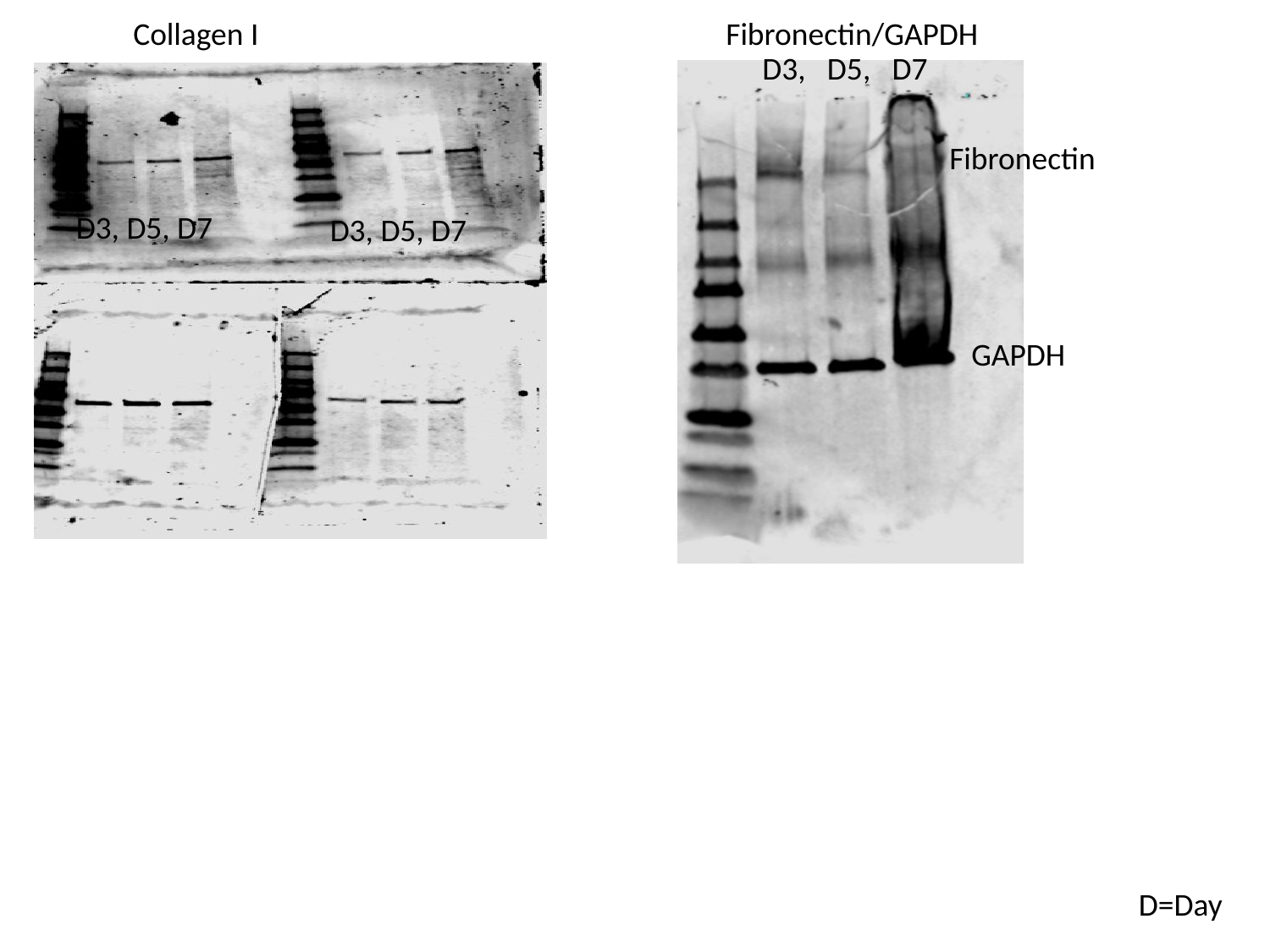

Collagen I
 Fibronectin/GAPDH
D3, D5, D7
Fibronectin
D3, D5, D7
D3, D5, D7
GAPDH
D=Day
